# The transcriptomic signature of DEPDC5 KO induced mTOR hyperactivation in human neurons and its response to rapamycin treatment

**DOI:** 10.1111/epi.18549

**Published:** 2025-07-24

**Authors:** Mattson S. O. Jones, Silvia Lindlar, Johannes Ludwig, Regina Waltes, Afsheen Kumar, Sophie v. Brauchitsch, Andrea Rossi, Evelyn Ullrich, Stefan Momma, Christine M. Freitag, Jasmin K. Hefendehl, Karl Martin Klein, Felix Rosenow, Denise Haslinger, Andreas G. Chiocchetti

**Affiliations:** ^1^ Autism Therapy and Research Center of Excellence, Department of Child and Adolescent Psychiatry, Psychosomatics and Psychotherapy, University Hospital Goethe University Frankfurt Frankfurt am Main Germany; ^2^ Center for Personalized Translational Epilepsy Research (CePTER) Goethe University Frankfurt Frankfurt am Main Germany; ^3^ Epilepsy Center Frankfurt Rhine‐Main, Center of Neurology and Neurosurgery Goethe‐University Frankfurt Frankfurt am Main Germany; ^4^ IUF‐Leibniz Research Institute for Environmental Medicine Düsseldorf Germany; ^5^ Experimental Immunology, Children's University Hospital Goethe University Frankfurt Frankfurt am Main Germany; ^6^ Frankfurt Cancer Institute (FCI) Goethe University Frankfurt Frankfurt am Main Germany; ^7^ German Cancer Consortium (DKTK), Partner Site Frankfurt/Mainz Frankfurt am Main Germany; ^8^ Institute of Neurology (Edinger Institute) Goethe University Frankfurt am Main Germany; ^9^ Institute of Cell Biology and Neuroscience University of Frankfurt Frankfurt am Main Germany; ^10^ Neurovascular Disorders, Buchmann Institute for Molecular Life Sciences University of Frankfurt Frankfurt am Main Germany; ^11^ Department of Clinical Neurosciences, Hotchkiss Brain Institute & Alberta Children's Hospital Research Institute, Cumming School of Medicine University of Calgary Calgary Alberta Canada; ^12^ Department of Medical Genetics, Hotchkiss Brain Institute & Alberta Children's Hospital Research Institute, Cumming School of Medicine University of Calgary Calgary Alberta Canada; ^13^ Department of Community Health Sciences, Hotchkiss Brain Institute & Alberta Children's Hospital Research Institute, Cumming School of Medicine University of Calgary Calgary Alberta Canada

**Keywords:** ASD, comorbidities, epilepsy, human cell model, neural progenitor cells, transcriptomics

## Abstract

**Objective:**

Mutations of the *DEP Domain Containing 5 gene* (*DEPDC5)*, a mechanistic Target of Rapamycin (mTOR) inhibitor involved in amino acid sensing, are associated with neurological diseases such as epilepsy and/or autism spectrum disorder (ASD). Loss of *DEPDC5* impacts early neuronal development via mTOR hyperactivity. Although, in the mTOR‐hyperactivity–associated syndrome tuberous sclerosis, mTOR inhibitors have proven to be beneficial in treating epilepsy, ASD‐associated symptoms are ameliorated only partially. Similarly, the mTOR inhibitor rapamycin (RAPA) only partially rescues phenotypes induced by loss of *DEPDC5* in animal models, suggesting some pathological mechanisms independent of mTOR.

**Methods:**

We dissected these mechanisms by identifying the *DEPDC5*‐associated gene networks and how they are targeted by RAPA in an isogenic primary human neural progenitor (phNPC) *DEPDC5* knock‐out cell model.

**Results:**

We confirm that loss of *DEPDC5* leads to hyperactivation of mTOR, paralleled by altered expression of mTOR‐associated genes. These effects were partially (up to 33% of genes) attenuated by RAPA treatment applying a clinically comparable concentration. We did not observe an association of the differentially expressed genes with ASD or epilepsy risk genes in general. However, we identified a significant association with gene networks known to be differentially regulated in cortex samples of individuals with ASD, which were still significantly deregulated after RAPA treatment. Furthermore, genes not rescued in differentiated neurons were specifically associated with synaptic pruning and early cortical development. The observed increase in neuronal markers was confirmed morphologically. RAPA treatment recovered the increased differentiation but not the morphological changes.

**Significance:**

These new insights on the human gene network of *DEPDC5* show evidence for pathological mechanisms that are not attenuated by the currently administered RAPA concentrations or that are independent of mTOR. These mechanisms should be considered as potential targets for future therapies.

**Key points**

- *DEP Domain Containing 5 DEPDC5*: Mutations in *DEPDC5*, a mechanistic Target of Rapamycin (mTOR) inhibitor, are linked to disorders such as epilepsy and autism spectrum disorder (ASD). Loss of *DEPDC5* results in mTOR hyperactivity, affecting early neuronal development.
- Partial Rescue by Rapamycin (RAPA): The mTOR inhibitor RAPA partially rescues the phenotypes induced by *DEPDC5* loss in animal models, indicating some pathological mechanisms are mTOR‐independent.
- Identification of *DEPDC5* Gene Networks and RAPA Treatment: In primary human neural progenitor cells, *DEPDC5*‐associated genes and networks were related to neuronal development, synaptic pruning, and p53 regulation.
- Disorder Association: Differentially expressed genes in the *DEPDC5* knock‐out model are associated with ASD and synaptic pruning, even after RAPA treatment.
- Pathological Mechanisms: Synaptic pruning and early cortical development‐related genes were not rescued in differentiated neurons with RAPA treatment, indicating mTOR‐independent mechanisms. These findings suggest new potential therapeutic targets beyond RAPA treatment and mTOR inhibition.

## 1 INTRODUCTION

Epilepsy and autism spectrum disorder (ASD) share a genetic etiology converging on pathways involved in neuronal communication and development.^1^ Among them is the mechanistic Target of Rapamycin (mTOR) pathway including inhibitory genes such as the *TSC complex subunit 1/2* (*TSC1/2*), *DEP Domain Containing 5* (*DEPDC5*), or *NPR2 Like 2/3* (*NPRL2/3*),^2^ the loss of which increases mTOR activation. Here, we investigated loss of mTOR inhibition at the messenger RNA (mRNA) level in primary human neural progenitor cells (phNPCs) and delineated the attenuation capacity of the mTOR inhibitor rapamycin (RAPA).

mTOR regulates cell size and protein translation^3^, ^4^ as well as amino acid sensing pathways. The latter is regulated by DEPDC5, NPRL2, and NPRL3 proteins within the GAP Activity TOward Rag 1 (GATOR1) complex. Loss of DEPDC5 leads to a constantly active mTOR‐kinase mimicking high amino acid concentration. Mutations of mTOR inhibitors have been identified in severe forms of epilepsy and ASD. Overall, 85% of all variants affecting GATOR1 are associated with familial focal epilepsy with variable foci (FFEVF) and over 80% of mutations are within *DEPDC5*.^5^ *DEPDC5* mutations have also been identified in ASD without epilepsy.^6^, ^7^ The penetrance of heterozygous *DEPDC5* mutations was estimated to 66%.^6^ However, more and more cases are reported where individuals carried a heterozygous germline *DEPDC5* mutation and an additional brain‐specific variant, thus resulting in a mosaic complete loss.^8^, ^9^, ^10^ DEPDC5‐associated mTOR hyperactivation has been published; however, the transcriptomic network changes at the neurodevelopmental level in human neurons are unknown.

In *DEPDC5*‐KO (knock‐out) models, including zebrafish,^11^ rat,^12^ and mouse,^13^, ^14^, ^15^, ^16^, ^17^, ^18^ mTOR hyperactivity and premature death, which was rescued by RAPA treatment, were reported consistently. In contrast, variable findings in heterozygous *DEPDC5* KOs indicate developmental differences between subspecies.^11^, ^15^, ^16^ Conditional KO (cKO) for specific brain regions resulted in dysmorphic neurons, altered brain sizes, and changes in cortex layer patterning, which recovered by early RAPA treatment.^13^, ^16^, ^17^ Knock‐down of *DEPDC5* and *NPRL3* in murine NPCs and neuroblastoma cells prevented mTOR co‐localization to the lysosome under amino acid starvation.^18^ mTOR hyperactivation, increased soma sizes, and increased rates of cell proliferation were replicated and attenuated through RAPA in human induced pluripotent stem cell (iPSC)‐derived neurons from heterozygous patients.^16^ One study in zebrafish investigated the brain transcriptome^10^ proposing an altered γ‐aminobutyric acid (GABA)ergic network upon loss of DEPDC5.

The RAPA derivative Everolimus is currently administered for the treatment of tuberous sclerosis complex (TSC)–associated epilepsy, reducing seizure frequency.^19^, ^20^ However, ASD phenotypes were not or only partially restored.^21^, ^22^, ^23^ Currently recommended therapeutic titration targets for the minimum plasma concentration in TSC treatment range between 5 and 15 ng/mL (5.2–15.7 nM)^24^ The highest maximum plasma concentration reported reaches up to 88 ng/mL (91.8 nM; pediatric patients).^25^

Here, we investigated the DEPDC5‐associated transcriptome network in isogenic proliferating and differentiating phNPCs. Specifically, we aimed at dissecting the mechanisms that can and cannot be rescued by 100 nM RAPA treatment and tested for association of these networks with ASD and epilepsy. Overall, this study aimed at investigating if RAPA is a potential treatment for *DEPDC5* mutation carriers and at emphasizing the non‐attenuated mechanisms as potential targets for additional therapies.

## 2 METHODS

For further details on methods, antibodies, primers, suppliers, and catalog numbers see Supporting Information.

### 2.1 Cell line cultivation

Primary human neural progenitor cells (phNPCs; D62) were cultured and propagated as neurospheres and grown as two‐dimensional culture as previously published.^26^ phNPCs were differentiated with retinoic acid, brain derived neurotrophic factor (BDNF) and Neurotrophin 3 (NT‐3) for 4 weeks and phNPC identity was confirmed using western blot, and immunocytochemistry (Figure S1). DEPDC5‐KO cells were treated with 100 nM RAPA (Absource Diagnostics): 18 h during proliferation and for 1 month during differentiation (for details see Methods S1).

### 2.2 Generation of DEPDC5 KO in phNPCs

D62 phNPCs cannot be expanded as single cells. Thus, all experiments were performed on a mixed KO cell population adapting a lentiCRISPRv2 (Plasmid #52961; Addgene^27^) strategy. In total, two small guidance sgRNAs (Dep2.1; and Dep2.2) targeting a patient‐specific mutation site (c.21C>G) in exon 2 of *DEPDC5* associated with ASD/epilepsy^6^ and one non‐targeting control (NTC) were designed and controlled for potential off targets. Lentivirus was produced in HEK293T. phNPCs were transduced with pure virus^28^ following puromycin selection for 5–7 days. Successful frameshifts in *DEPDC5* exon 2 were confirmed via Sanger sequencing (Figure S2A,B).

#### 2.2.1 Western blot

For (phospho‐) protein detection we did semi‐dry blotting (Trans‐Blot Semi‐Dry Transfer Cell; Bio‐rad) on a polyvinylidene difluoride (PVDF) membrane (Immobilon‐FL) with secondary antibodies coupled to horseradish peroxidase. Blots were stripped between stains, reblocked, and re‐stained. For further details on antibody dilutions and imaging see Methods S1.

### 2.3 Functional characterization of DEPDC5 KO vs NTC

#### 2.3.1 Immunocytochemistry

phNPCs were seeded directly on coverslips and cultured as described. After fixation, cells were submerged in 20 μL primary antibodies (1:500 dilution) overnight following a 1:1000 secondary antibody incubation. Coverslips were mounted with Prolong Gold Antifade with DAPI (Thermo Fisher). Cells were visualized with a Nikon Eclipse Ti Confocal Microscope equipped with a spinning disc unit (CSUW1, Andor) and LED lasers at wavelengths 405, 488, 561 and 639 nm. Visualization was done with Nikon Elements software (version 4.60.00 Build 1171).

#### 2.3.2 Starvation assay

A total of 2–3 × 10^6^ proliferating cells were incubated in 10 cm cell culture dishes for 90 min in Neurobasal A or Neurobasal A without amino acids (US Biological) supplemented with 3.7 g/L sodium bicarbonate (Gibco). For rescue experiments, cells were incubated with 100 nM RAPA (Absource Diagnostics). Immediately following incubation, cells were lysed in RIPA buffer. Protein extracts underwent western blotting as described.

#### 2.3.3 Transcriptomic analysis (3′‐mRNA sequencing)

RNA was extracted from three biological replicates of NTC, Dep2.1, and Dep2.2 with or without 100 nM RAPA (18 h of RAPA on proliferating cells; 1 month during neuronal differentiation), respectively. All samples passed quality check with an RNA integrity number (RIN) >7. RNA libraries were generated with Quantseq 3′‐mRNA Library Prep (Lexogen) and sequenced on a HiSeq 2500 V4 (High Output Mode) with a coverage of 10 M Raw Reads per sample and a standard read length of 1 × 50 bp (NGS Core Facility Bonn). Raw reads were quality controlled (FastQC; https://www.bioinformatics.babraham.ac.uk/projects/fastqc/), trimmed (trimmomatic^29^), and aligned to hg38 (Rsubread^30^) using standard settings. Feature counts were extracted and used for subsequent statistical analysis (see subsequent text).

#### 2.3.4 Morphology and cell counting

Analysis of cell sizes as well as neuronal, astrocyte, and progenitor counts were done using FIJI (v. 1.53f51). For cell counts, three images of 100 × 100 μm from different slides were analyzed per condition. Sholl analysis of MAP2‐stained cells was performed by counting 20 cells per condition with the Simple Neurite Tracer (version 4.0.3) with a radius step of 1, semi‐log method, and “Best fitting” degree settings. These 20 cells were imaged across three technical replicates per condition.

### 2.4 Statistical analysis

#### 2.4.1 RNA‐sequencing analysis

Full code and results of RNA Sequencing data analysis is available online as R‐markdown output https://kjpmolgenlab.github.io/CePTER_RNASeq/index.html.

Non‐unique annotations (i.e. reads mapped to different gene isoforms) were merged by sum. Technical sample outliers (normalized counts per million (cpm) distribution and clustering based), and genes with no variance or genes detected in less than 50% of the samples were excluded. (Average read per sample; 7 195 819 (standard deviation SD: 345374) and 13 731 genes passing QC). Quality control showed clear separation of samples by differentiation stage, with no technical outliers (DIFF, PROLIF, Figure S3A–C). Count normalization and the statistical analysis were performed using DESeq2 with the full model being:

$$\begin{array}{c} \mathrm{cpm}\sim \text{intercept}+\text{factor}\left(\text{gRNA}\right)+\text{factor}\;\left(\text{DIFF}\right)\\ \;+\text{factor}\;\left(\text{RAPA}\right) \end{array}$$

Differential expression (DEX) was estimated using “DESeq” with standard options. Genes were considered to be significant if the FDR was <.05 with the same direction of the log2FC in both KO cell lines compared to the NTC control, respectively. Resulting deregulated genes were analyzed in the KEGG Mapper Tool (https://www.genome.jp/kegg/mapper/search.html), Entrez gene database summary (https://www.ncbi.nlm.nih.gov/gene), or Genecard database summary (https://www.genecards.org/). Significant genes were further investigated for enrichment among ASD and epilepsy genes and biological processes (see subsequent text).

#### 2.4.2 Weighted gene co‐expression network analysis (WGCNA)

We followed the standard recommendation of the WGCNA package in R^31^ (softPower = 6; Scale free topological model fit > 90%, scree plot of Mean connectivity; min. genes within a set = 50). Module Eigengenes were calculated. Modules were merged if they clustered together with a distance < 0.2. Differences between KO and NTC were tested as described, correcting *p*‐values (*corP*) for the number of modules tested.

#### 2.4.3 Gene list enrichment testing

DEX genes were Fisher‐exact tested for enrichment among high‐risk genes for epilepsy (Epi25 https://epi25.broadinstitute.org/ ^32^, ASD https://gene.sfari.org/,^33^, ^34^, ^35^ Fragile X syndrome,^36^ TSC,^37^, ^38^ intellectual disability (ID),^39^, ^40^ or schizophrenia^41^, ^42^; see Table S2). Gene lists were considered as associated with DEPDC5‐KO if the odds ratio (OR) was >1 and the Bonferroni adjusted *p*‐value (corP) < .05 (Fisher exact test). The full gene lists are available as Table S1. Gene‐ontoloty (GO) term enrichment analysis was performed implementing the gprofiler2^43^ package (correction_method=”g_SCS”). All genes passing quality control in our analysis were set as a reference gene universe.

To evaluate the developmental impact of the KO of DEPDC5, DEX genes were analyzed in the MAGNET pipeline (MApping the Genetics of neuropsychiatric traits to the molecular NETworks of human brain pipeline, https://molgenlab.shinyapps.io/MAGNET_lite_V2/ ^44^). This in‐house pipeline is based on the Allen Brain Atlas^45^ using 1340 tissue samples of 57 postmortem brains. DEX genes were tested with Fisher exact test for significant enrichment among the 29 co‐regulated modules during neurodevelopment as published^44^, ^45^ (*corP* < .05).

For estimating the overall attenuation effects, we implemented linear mixed regression models.

#### 2.4.4 Morphology

Group differences for the morphological analysis were tested using the two‐sample unequal variance *T* test, where significance was defined if *p* < .05. All samples were compared to their respective controls (NTC vs Dep2.1 or Dep2.2). Mean measurements and standard error were calculated for each morphological parameter.

## 3 RESULTS

### 3.1 DEPDC5 KO increases mTOR activity in phNPCs

Overall, the D62 cell line displayed characteristics of neural progenitor cells during proliferation. MAP2‐positive neurons, as well as astrocytes and NPCs, were detected after 4 weeks of differentiation (Figure S1). We successfully induced DEPDC5 KOs (2 KO cell lines: Dep2.1 and Dep2.2), confirmed via Sanger sequencing and western blot analysis (Figure S2). Quality control based on RNASeq data confirmed replicability of differentiation and KO across biological and technical replicates (Figures S3 and S4A). In all cell lines, *DEPDC5* mRNA was detected, suggesting that the KO occurs during translation, where a premature stop codon leads to disruption of protein synthesis (Figure S4A).

We further confirm mTOR hyperactivation via increased phosphorylation of S6 (pS6) and decreased pAKT, indicating that the reverse feedback mechanism is intact.^13^ This hyperactivity was attenuated by RAPA (Figure S2D). Taken together, we successfully knocked out *DEPDC5* in D62 phNPCs and confirmed hyperactivation of mTOR.

### 3.2 *DEPDC5* KO affects cell cycle and differentiation

#### 3.2.1 Gene module analysis

WGCNA identified seven independent co‐regulated gene modules (Table S2) across all samples.

**FIGURE 1:**
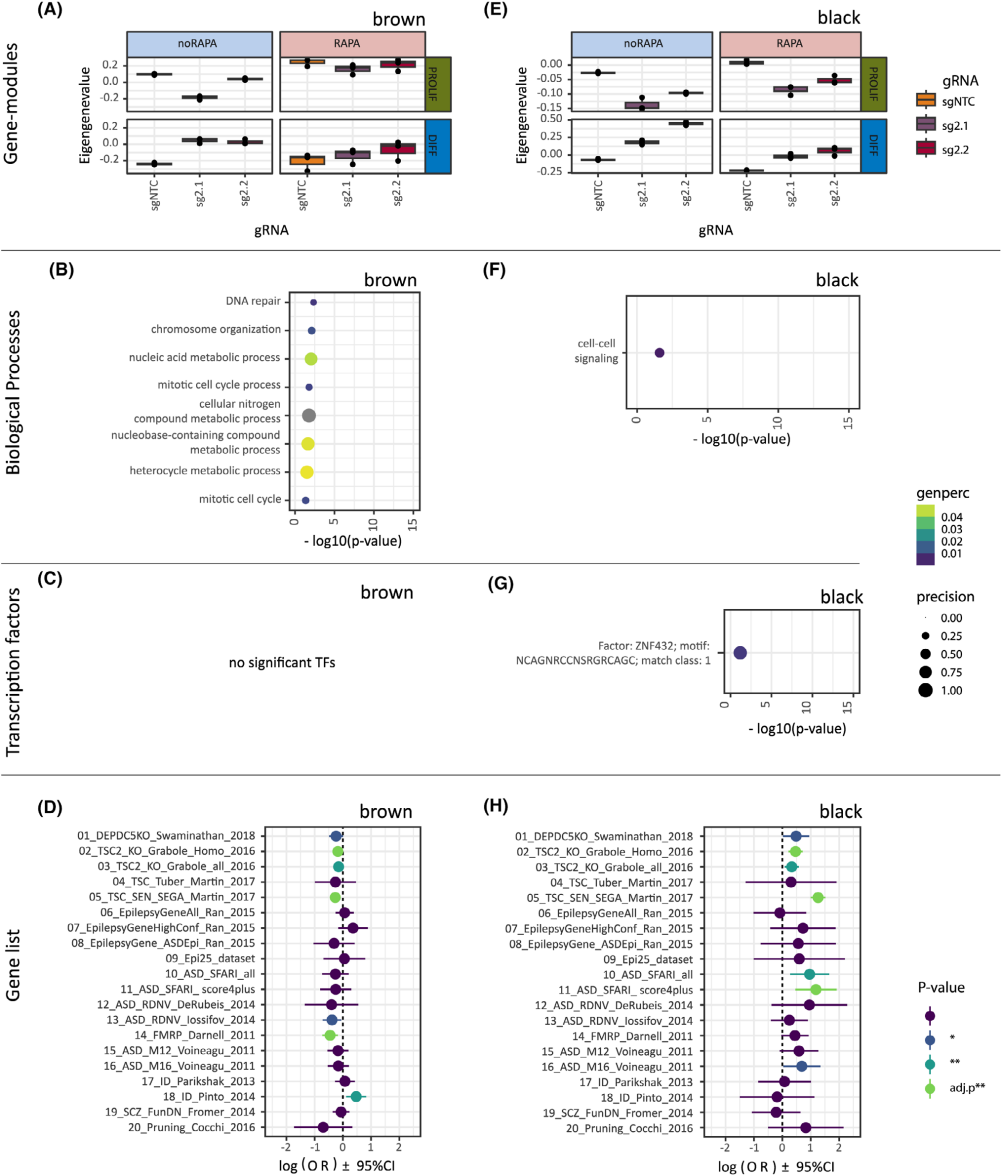
Significantly replicated modules identified by weighted gene co‐expression network analysis (WGCNA) during proliferation (PROLIF) and differentiation (DIFF). (A) Eigengene values of the WGCNA brown module during proliferation and differentiation, with and without Rapamycin (RAPA) treatment. (B) Gene ontology (GO) terms associated with the brown module. (C) Transcription factor (TF) enrichment analysis for the brown module. (D) Gene list (Table S2) enrichment analysis for the brown module. Adjusted *p*‐values are Bonferroni‐corrected. (E) Eigengene values of the black WGCNA module during proliferation and differentiation, with and without RAPA treatment. (F) GO terms associated with the black module. (G) Transcription factor analysis of the black module. (H) Gene list (Table S2) enrichment analysis for the black module. Abbreviations: ASD, Autism Spectrum Disorder; Conf, Confidence; Epi, epilepsy; FunDN, functional de‐novo; ID, Intellectual Disability; RDNV, Rare‐denovo; SFARI, Simons Foundation Autism Research Initiative; SCZ, Schizophrenia; TSC, Tuberous Sclerosis Complex.

Statistical analysis identified two significantly regulated gene modules in proliferating cells (Figure 1A–D): the Brown module (related to translation, cell cycle, and mitosis) and the Black module (related to cell–cell signaling). The Eigengene values of these modules were significantly downregulated upon DEPDC5 KO compared to NTC (Brown module: corP < .01; Black module: corP < .01). Specifically, the downregulation in the Brown module suggests impaired cell cycle regulation and protein translation processes, which are directly linked to mTOR signaling. The Black module was also downregulated, highlighting potential disruptions in early cellular interactions critical for neuronal development.

Both modules responded to RAPA treatment, showing partial restoration toward untreated NTC levels, particularly for cell cycle–related processes in the Brown module. This finding, as expected, supports a direct role of mTOR signaling in the proliferation‐associated phenotypes observed in DEPDC5 KO cells.

In differentiating cells, the pattern reversed: Both the Brown and Black modules showed significant upregulation following DEPDC5 KO (Brown module: corP < .01; Black module: corP < .01). Although the Brown module is significantly downregulated in DIFF vs PROLIF untreated NTC condition, the increased activation upon DEPDC5 KO in DIFF indicates enhanced translation and cell cycle–associated activity despite differentiation conditions. This is suggesting an abnormal persistence of proliferative mechanisms. In contrast, the Black module's Eigenvalue in NTC is similar on both DIFF and PROLIF, respectively. However, the increased expression upon KO during differentiation highlights heightened neuronal communication and cell signaling activities, potentially reflective of compensatory or maladaptive responses to early developmental disruptions.

Similar to proliferation conditions, RAPA treatment in differentiating cells attenuated the alterations in the Brown module, confirming its strong mTOR dependence. However, changes in the Black module were only partially rescued by RAPA, indicating the involvement of additional mTOR‐independent regulatory mechanisms during neuronal differentiation or that clinically administered RAPA doses may be insufficient for full rescue.

Taken together, both modules were responsive to RAPA treatment in that the treatment attenuated the *DEPDC5* KO effect toward untreated D62‐NTC cell lines. We also observed an increased activity of the Brown module in the NTC‐RAPA compared to the NTC‐noRAPA cells. Although this is counterintuitive, as mTOR inhibition would be expected to lead to a reduced cell‐division, we attribute this change to compensatory mechanisms at the mRNA level, or simple batch effects.

To identify potential targets for regulation of these to modules and to understand their disease relevance, transcription factor (TF) and gene list enrichment analyses were performed (Figure 1E–H). Although no significant enrichment for a TF motif was identified for the Brown module (Figure 1E), we report a significant association of the Black module with the ZNF432 motif (Figure 1F). The TF ZNF432 itself was not differentially expressed upon *DEPDC5* KO (corP > 0.9 in both KOs). Of interest, the ASD‐associated genes, *NRXN1* and *NRXN2*, as well as the inhibitory receptor *GABRB3* and the GABA synthesizing enzyme *GAD2* were the most prominent candidates within the Black module that were also targets of ZNF432 (Table S2). With respect to disease relevance, the Brown module only showed nominal enrichment for ID‐associated genes (Figure 1G), whereas the genes of the Black module were significantly enriched for TSC‐associated genes as well as for high‐evidence ASD candidate genes (Figure 1H). This highlights the Black module as a central component within the etiological mechanisms.

#### 3.2.2 Transcriptomic analysis

**FIGURE 2:**
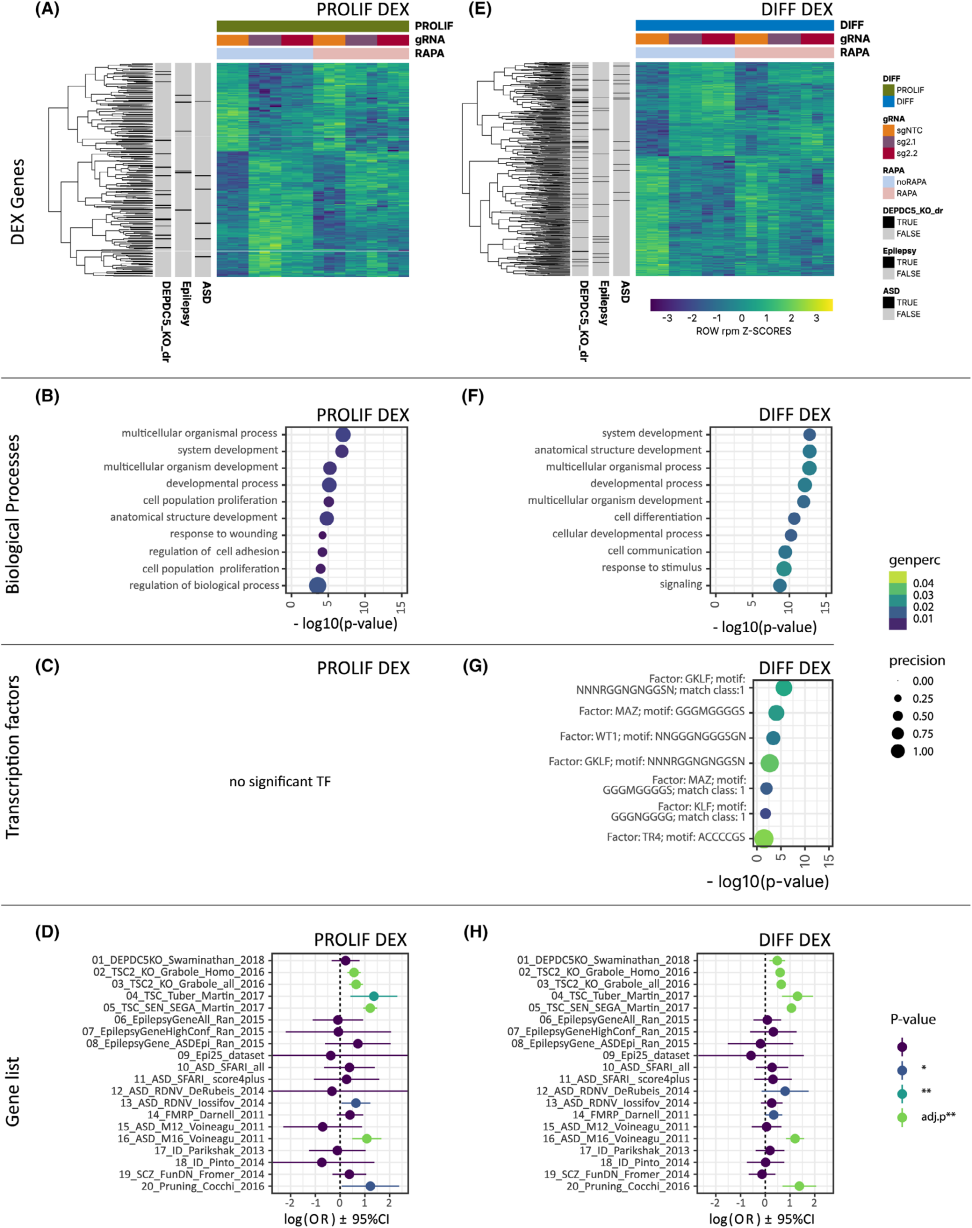
Genes differentially expressed upon DEPDC5‐KO during proliferation and differentiation. (A) Heatmap of differentially expressed genes in proliferation state (PROLIF‐DEX). Gene lists: 01/DEPDC5_KO_dr, 06/Epilepsy, 10/ASD. (B) GO terms associated with PROLIF‐DEX genes. (C) Transcription factor enrichment analysis for PROLIF‐DEX. (D) Gene list (Table S2) enrichment analysis for PROLIF‐DEX. Adjusted *p*‐values are Bonferroni‐corrected. (E) Heatmap of differentially expressed genes in differentiation state (DIFF‐DEX). Gene lists: 01_DEPDC5KO_Swaminathan_2018, 06_EpilepsyGeneAll_Ran_2015, 10_ASD_SFARI_all. (F) GO terms associated with DIFF‐DEX genes. (G) Transcription factor analysis for DIFF‐DEX. (H) Gene list enrichment analysis for DIFF‐DEX. Colored dots represent significance for uncorrected *p*‐values. Green dots mark enrichments that were also significant after correction for multiple testing. Abbreviations: ASD, Autism Spectrum Disorder; Conf, Confidence; Epi, epilepsy; FunDN, functional de‐novo; ID, Intellectual Disability; RDNV, Rare‐denovo; SFARI, Simons Foundation Autism Research Initiative; SCZ, Schizophrenia; TSC, Tuberous Sclerosis Complex.

At the single gene level, we observed a total of 237 DEX genes during proliferation (PROLIF‐DEX, 139 upregulated, 98 downregulated, Figure 2A; Figure S4B; Tables S4 and S5). These genes were enriched for the GO terms “developmental processes” and “cell adhesion” (Figure 2B), among them the mTOR‐targeted Ribosomal Protein S6 gene (*RPS6* avg. log2FC = −3.34E‐01, corP < .05) or the downstream effector of the mTOR pathway, the Cyclin Dependent Kinase Inhibitor 1A/P21 (*CDKN1A* avg. log2FC = 1.32, corP < 5.94E‐32) as well as the MDM2 Proto‐Oncogene, a negative regulator of P53, another downstream effector of cell cycle regulation of mTOR (*MDM2* avg. log2FC = .517, corP < .01). Protein–protein interaction and clustering analysis of the PROLIF‐DEX identified six clusters (C1–C6) with more than five interaction partners. These clusters were enriched for genes associated with Cell cycle (C1), Cell Migration (C2), TP53 Network (C3), Cytoskeleton formation (C4), Nervous system development (C5), and Neuron Projection (C6) (Figure S5A,B).

We did not observe significant enrichment of PROLIF‐DEX genes among genes previously associated with *DEPDC5* KO in zebrafish^11^ (Figure 2D; Table S5). However, we observed a significant overlap with genes previously published^37^, ^38^ in the Tuberous Sclerosis mouse models (all odds ratios ORs > 1.70, corP < .01) and with genes belonging to a gene‐network differentially regulated in ASD^35^ (OR = 1.63–4.96 corP = 5.61E‐03; Figure 2D). Nominal enrichment (OR = 1.06–3.17, uncorP = 2.25E‐02) was found for ASD‐associated genes identified via de novo sequencing analyses^34^—among them *NRXN3* (avg log2FC = −1.403, corP < .001), one of the most discussed candidate genes for ASD.^46^ We identified no significant enrichment for epilepsy risk genes among the PROLIF‐DEX genes (Figure 2D). We further highlight a significant downregulation of the developmental and epileptic encephalopathy (DEE) associated gene *KCNQ2* (potassium voltage‐gated channel subfamily Q member 2; avg. log2FC = −.615 corP < .002) as well as the spectrum repeat containing nuclear envelope protein 2 gene *SYNE2* (avg log2FC = −1.059, corP < 6.121E‐5).

In differentiating cells, a total of 570 genes (DIFF‐DEX, 250 upregulated, 320 downregulated, corP < .05, Figure 2E; Figure S4B; Table S4) were found to be DEX. Of those, 51 genes were already detected in the proliferation stage, including *NPY* (Neuropeptide Y), *SERPINE2* (Serpin Family E Member 2), and *STMN2* (Stathmin 2). Among the DIFF‐DEX genes, 27 were associated with mTOR (e.g., *castor1* avg. log2FC = −1.21, corP < .05; Table S4). Again, the MDM2 Proto‐Oncogene was among the DIFF‐DEX genes (*MDM2* avg. log2FC = −.828, corP < 7.93E‐6) as well as *CDKN1A* (avg log2FC = −.892, corP < 2.96E‐9).

We observed enrichment for extracellular matrix formation, neuronal development, and differentiation (Figure 2F). Overall, the DIFF‐DEX genes were associated with the TF motif for the P53 (cell cycle regulator and tumor suppressor) associated TF GKLF (KLF4), WT1, and the 16p11.2 deletion syndrome‐associated TF MAZ (Figure 2G). Protein–protein interaction and clustering analysis of the DIFF‐DEX identified 26 clusters with more than 5 interaction partners. The top five clusters by size (C1–C5) were associated with Translation (C1), Cell Signaling (C2), Extra Cellular Matrix Formation (C3), Splicing (C4), and Junction Assembly (C5; Figure S5C,D).

DIFF‐DEX genes significantly overlapped with the genes identified in *DEPDC5* KO zebrafish (OR = 1.18–2.19, corP = 4.94E‐02), with the ASD‐associated gene network and association (OR = 2.32–4.69, corP 1.51E‐08), as well as with the TSC genes (all OR > 1.8, corP < 1.06E‐03; Figure 2H). In addition, nominal enrichment (OR = 1.0–1.94; uncorP = 4.00E‐02) was detected for genes targeted by the ASD‐associated FMRP (Fragile X Mental Retardation Protein) as well as genes preferentially hit by rare mutations in ASD (^33^; OR = .86–4.86, uncorP = 4.81E‐02). We further report an upregulation of *NRXN3* (avg log2FC = 1.809 corP < .026) as well as *NRXN1* (avg lo2FC 3.803, corP < 1.22E‐4; Table S4). No significant overall enrichment with epilepsy risk genes was observed. However, single epilepsy risk genes such as the ionotropic glutamate receptor *GRIN2A* (avg log2FC = 2.25 corP < .003), Doublecortin (*DCX*, avg. log2FC = 2.405, corP < 1.81E19), or the RAN Binding Protein 17 gene *RANBP17* (avg log2FC = 6.746, corP < .04) were differentially expressed.

**FIGURE 3:**
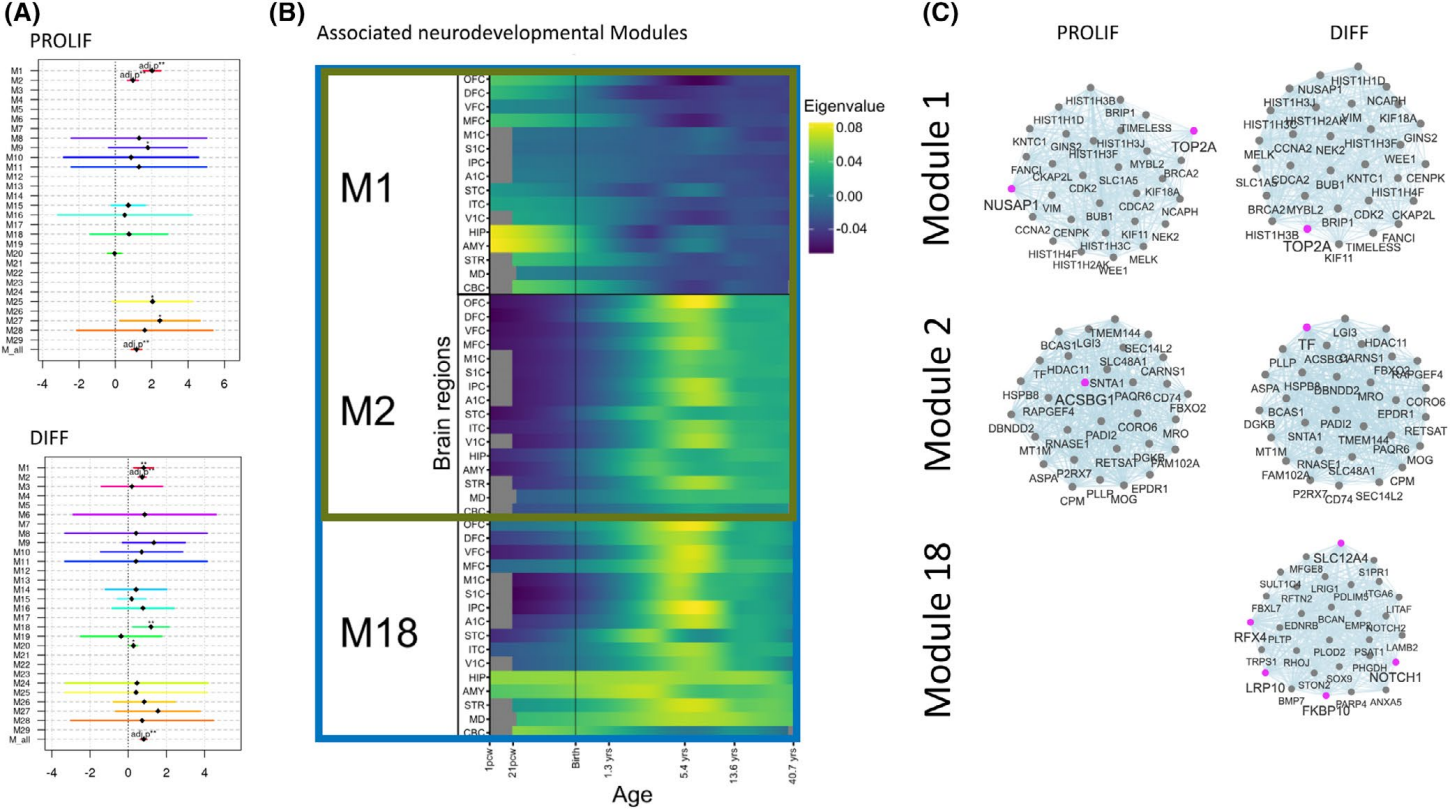
Spatially and temporally expressed genes corresponding to DEPDC5‐KO using the Kang dataset. (A) Kang modules enriched for PROLIF‐DEX or DIFF‐DEX genes. ** adj. *p*‐value. (B) The associated developmental networks correspond to Modules 1 and 2 for both proliferation (red square) and differentiation (blue square) DEPDC5 KO expressed genes, while Module 18 is differentiation specific (Kang et al. 2011). A1C, Primary auditory (A1) cortex; AMY, amygdala; DFC, dorsolateral prefrontal cortex; HIP, hippocampus; IPC, posterior inferior parietal cortex; ITC, inferior temporal cortex; M1C, primary motor (M1) cortex; MFC, medial prefrontal cortex; OFC, orbital prefrontal cortex; S1C, primary somatosensory (S1) cortex; STC, superior temporal cortex; STR, striatum; V1C, primary visual (V1) cortex; VFC, ventrolateral prefrontal cortex. (C) Hub gene networks developed for the three identified modules in proliferation and differentiation datasets. Only adjacency > .4 are shown to depict network density.

Finally, to understand the developmental impact of DEX genes in the PROLIF and DIFF condition, respectively, we tested for enrichment of developmental gene modules previously identified. PROLIF‐DEX genes were enriched for genes in brain developmental Modules 1 and 2, whereas DIFF‐DEX genes were associated with Modules 1, 2, and 18 (Figure 3A). Modules 1 and 18 are activated during early hippocampus and amygdala development, whereas Module 2 is associated with later developmental stages in the striatum and cerebral cortex (Figure 3B). The DEX hub‐gene in Module 1 was *TOP2A*, differentially expressed in both PROLIF and DIFF. In Module 2, *ACSBG1* (PROLIF‐DEX) and *TF* (DIFF ‐EX) were most central. Finally, for Module 18, the highly connected genes *SLC12A4*, *FKBP10*, *RFX4*, *BMP7*, *NOTCH1*, and *LRP10* were DEX‐DIFF (Figure 3C).

### 3.3 Rapamycin rescues cell cycle and cell communication but not neuronal development, differentiation, and pruning

**FIGURE 4:**
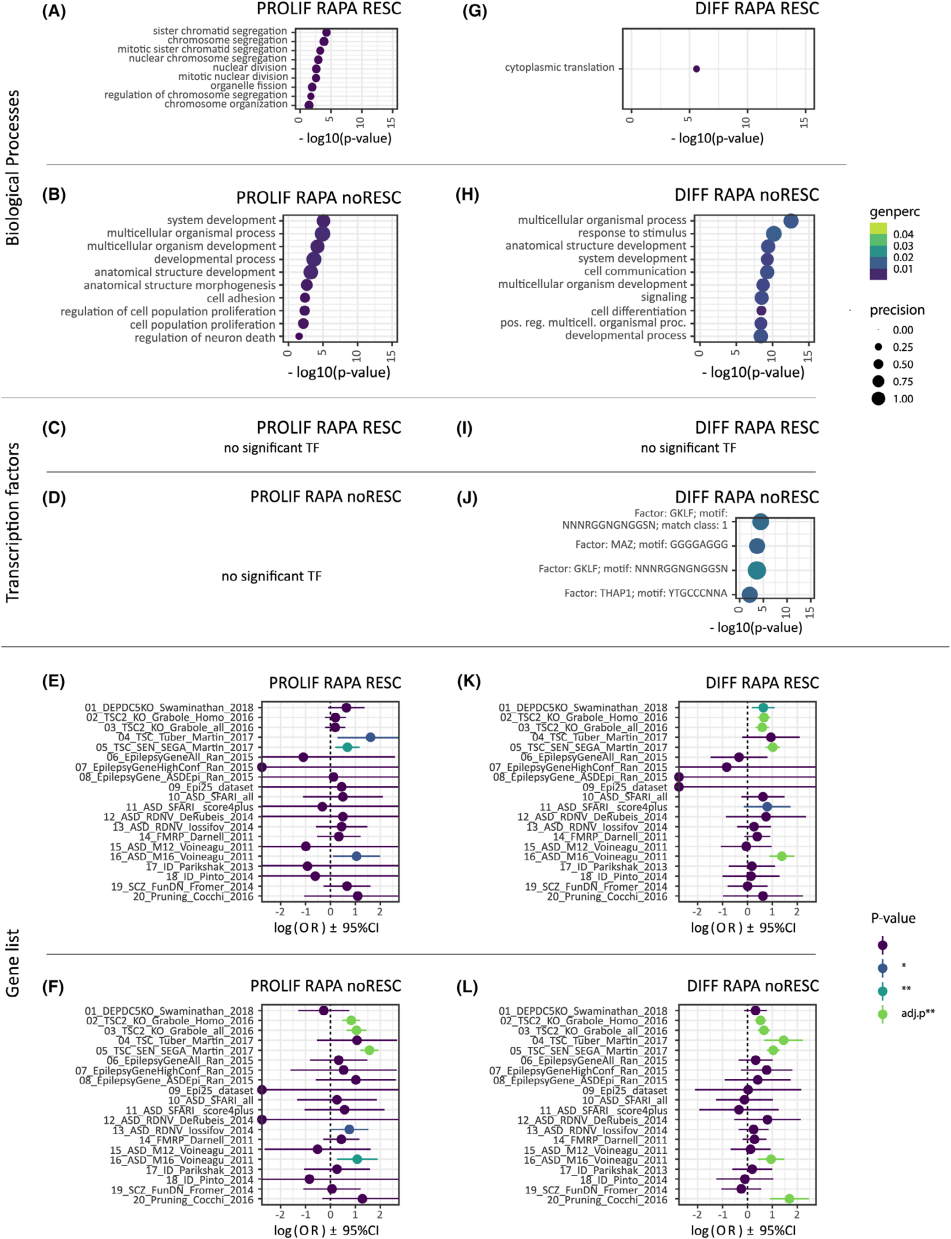
Biological pathways rescued vs not rescued by RAPA treatment of DEPDC5 KO during proliferation and differentiation. (A) GO terms associated with PROLIF‐DEX genes rescued by RAPA. (B) GO terms associated with PROLIF‐DEX genes NOT rescued by RAPA. (C) Transcription factor enrichment analysis for PROLIF‐DEX rescued by RAPA. (D) Transcription factor enrichment analysis for PROLIF‐DEX not rescued by RAPA. (E) Gene list (Table S2) enrichment analysis for PROLIF‐DEX rescued by RAPA. (F) Gene list enrichment analysis for PROLIF‐DEX not rescued by RAPA. (G) GO terms associated with DIFF‐DEX genes rescued by RAPA. (H) GO terms associated with DIFF‐DEX genes not rescued by RAPA. (I) Transcription factor enrichment analysis for DIFF‐DEX rescued by RAPA. (J) Transcription factor enrichment analysis for DIFF‐DEX not rescued by RAPA. (K) Gene list (Table S2) enrichment analysis for DIFF‐DEX rescued by RAPA. (L) Gene list enrichment analysis for DIFF‐DEX not rescued by RAPA Colored dots represent significance for uncorrected *p*‐values. Green dots mark enrichments that were also significant after correction for multiple testing.

We here defined RAPA attenuation if a DEX gene was not any more significantly expressed between untreated NTC and the treated KO (corP >.05) or if the direction of the effect was reversed (e.g., overshoot of effect). In general, we observe that RAPA attenuated the effect of *DEPDC5* KO in proliferating (beta = .533, *p* < .001) as well as in differentiating cells (beta = .728, *p* < .001); of note a linear regression coefficient beta >0 and <1 indicates an overall shift of the log2FC toward 0 in the treated cells. Of the 237 PROLIF‐DEX genes, 162 genes were rescued (Table S4, Figure S4B). These were associated with cell cycle and division (Figure 4A). The 75 genes that remained significant were associated with neurite outgrowth and development (Figure 4B; Table S5), among them the mTOR gene *RPS6* (avg log2FC −.444, corP < .015). We did not identify any significant TF enrichment among rescued and non‐rescued PROLIF‐DEX genes (Figure 4C,D).

We observed that the PROLIF‐DEX genes rescued by RAPA were only nominally enriched for one TSC list (Figure 4E). However, PROLIF‐DEX genes not rescued by RAPA show significant enrichment for TSC2‐associated genes (Figure 4F). With respect to the down‐regulated ASD gene *NRXN3*, the rescue was inconclusive, as it was still significantly downregulated in one KO but not in the other (log2FC_2.1_–1.44 corP = 2.943E‐4; log2FC_2.1_–.762 corP = .222). The epilepsy risk gene *KCNQ2* was not rescued (avg log2FC −.763 corP < .01), whereas *SYNE2* was (corP > .675).

A total of 381 of the 570 DIFF‐DEX genes were rescued by RAPA treatment (Figure S4B). At the functional level, RAPA treatment rescued cell–cell communication and protein synthesis but not neuronal development and differentiation (Figure 4G,H; Table S5). Here, we observed that non‐rescued DIFF‐DEX genes were enriched for transcription factor motifs for GKLF (KLF4) (corP = 2.01E5), KLF (corP = .029), WT1 (corP = .003), and MAZ (corP = .003) (Figure 4I,J). Testing DIFF‐DEX for enrichment with disease risk genes, we do still see an enrichment for both rescued and non‐rescued genes, with *TSC2* genes, or with the gene set related to ASD brain modules (Figure 4K,L). Genes associated with *DEPDC5* KO in zebrafish show only nominal enrichment for the RAPA‐rescued genes (Figure 4K), whereas synaptic pruning genes were significantly enriched for genes not rescued by RAPA treatment. Of interest, 60% of the non‐rescued DIFF‐DEX genes in the pruning gene list showed a higher expression in the KOs.

The DIFF‐DEX ASD genes *NRXN1* and *NRXN3* both were rescued by RAPA treatment (corP > .227). The epilepsy risk gene *GRIN2A* was not rescued and remained upregulated (avg log2FC = 2.664, corP < 2.36E4). RANBP17 still showed a strong upregulation with a trend toward significance (log2FC_2.1_ = 6.133, corP = .064, log2FC_2.2_ = 7.142, corP = .002). However, for DCX, the results were inconsistent for the two KO cell lines (log2FC_2.1_ = −.403; corP = .490; log2FC_2.1_ = .868, corP = .011).

Overall, we confirm that the KO of DEPDC5 can be partially attenuated by RAPA treatment, while at the same time loss of DEPDC5 affects genetic networks and risk genes that are insensitive to RAPA.

### 3.4 Morphological analysis confirms transcriptome data

We observed a significantly (*p*‐value = .0049) higher percentage of MAP2‐positive neurons in the KO cells, which was attenuated by RAPA treatment (Figure S6A,B). We further observed a significant reduction of the average neurite length, paralleled by an increased complexity (dendritic branching), which were not restored by RAPA treatment (*p*‐value < .5) (Figure S6C,D).

## 4 DISCUSSION

Overall, we confirm that loss of DEPDC5 induces a hyperactivation of mTOR, corroborating previous studies.^13^, ^17^ This hyperactivation is closely linked to translational changes, thus implying that the observed effects are secondary consequences of mTOR dysregulation. Treatment with RAPA reversed mTOR hyperactivation, restoring increased phosphorylation of RPS6 and partially correcting the differential expression related to cell cycle regulation and cell–cell signaling. Here, RAPA treatment restored mTOR activity to untreated NTC conditions. The persistent pRPS6 levels as observed here are in line with a reduced RAPA concentration compared to previous studies,^11^, ^12^ completely blocking mTOR. The limited rescue of observed changes, as reported here, warrants consideration of RAPA treatment dose and duration, as well as targeting the specific mechanisms reported here in future studies.

### 4.1 Proliferating cells

In PROLIF phNPCs, the significant downregulation of the Brown and Black modules following DEPDC5 KO suggests disruptions in cell cycle regulation, protein translation, and initial cellular interactions essential for neuronal development. The downregulation of the Brown module corresponds with established mTOR functions such as protein synthesis and cell cycle progression,^47^ confirming that DEPDC5 loss impairs essential processes during early neuronal development. Similarly, reduced expression in the Black module indicates impaired neuronal communication and cell adhesion, which may contribute to disrupted early neuronal network formation. Partial restoration of these modules with RAPA highlights the direct involvement of mTOR in these processes. However, incomplete rescue might indicate that the dose of 100 nM or 96 ng/mL (clinically max concentrations are below 88 ng/mL^25^) is not fully effective in attenuating the pathological mechanisms relevant for neuronal development. However, the restoration of mTOR hyperactivity to wild‐type levels, as observed for the Brown mTOR‐associated module and in the western blot results, fosters the hypothesis of additional mTOR‐independent pathways contributing to pathological mechanisms during the proliferation stage.

### 4.2 Differentiating cells

In DIFF‐phNPCs, we observed a reversal of the modules' expression patterns, characterized by significant upregulation of both Brown and Black modules, indicating complex adaptive or maladaptive responses following DEPDC5 loss. Persistent activation of the Brown module, despite differentiation signals, suggests abnormal continuation of proliferative and translational processes. This persistent activity likely disrupts normal neuronal maturation, possibly explaining the observed morphological abnormalities. Increased activity in the Black module during differentiation further underscores disturbances in neuronal communication, potentially reflecting compensatory responses to developmental deficits established earlier. Again, the incomplete rescue by RAPA in these cells suggests contributions from mTOR‐independent mechanisms and/or reduced efficiency of clinically administered RAPA concentrations,^25^ particularly affecting synaptic connectivity and signaling. Overall, the distinct patterns of gene module dysregulation between proliferation and differentiation stages provide critical insights into the dynamic and complex nature of DEPDC5‐associated pathology.

### 4.3 Comparative insights across developmental stages

Although the observed changes provide important insights into the potential mechanisms underlying *DEPDC5* pathology, caution is necessary when translating these findings directly to human ASD pathology. The human neural progenitor cell model used in this study represents early developmental stages, and direct correlations to clinical outcomes in ASD patients remain speculative. Furthermore, therapeutic effectiveness observed in cell models might not precisely predict clinical efficacy in postnatal or adult treatments due to the developmental timing differences and complexities inherent in human neuronal development.

However, the distinct gene module dysregulation patterns between proliferation and differentiation stages underscore the dynamic and complex nature of DEPDC5 pathology. Although proliferating cells show impairments closely linked to mTOR‐dependent processes, differentiating cells are likely to exhibit additional dysregulation driven by compensatory or secondary pathological mechanisms. These findings emphasize that therapeutic interventions targeting mTOR alone, particularly at later developmental stages or adulthood, may not sufficiently mitigate neurodevelopmental effects of *DEPDC5* mutations. Future strategies should thus address both mTOR‐dependent and mTOR‐independent mechanisms comprehensively.

### 4.4 Individual gene‐level observations

At the individual gene level, we observed enrichment for genes involved in protein and ribosome biogenesis, endoplasmatic reticulum (ER) membrane transport, p53‐dependent cell cycle control, nucleotide and lipid metabolism, and protein degradation—all processes known to intersect with mTOR regulation. Although we did not identify complete dysregulation of mTOR pathway components at the gene level, several key downstream and upstream targets were significantly affected.

Of interest, PROLIK KO cells exhibited decreased *RPS6* mRNA levels, despite increased protein phosphorylation, suggesting complex feedback mechanisms at the transcriptional level. As literature addressing transcriptional feedback loops involving RPS6 mRNA remains limited, further investigation is warranted. In addition, we identified notable effects on ASD‐associated genes *NRXN3* and *NRXN1*, consistent with observations in TSC1/2 human neuronal stem cell models.^48^ Thus, *DEPDC5* mutations may influence ASD pathology through altered synaptic receptor regulation and cell adhesion mechanisms, pathways prominently implicated in ASD.^47^

### 4.5 Synaptic pruning and mTOR‐independent mechanisms

In this study, which applied a clinically comparable dose, several genes affected by DEPDC5 KO were resistant to rescue by rapamycin (or RAPA) particularly, in differentiating (or DIFF) cells. Genes associated with synaptic pruning remained significantly dysregulated. Previous animal models, such as the TSC2 model, reported increased dendritic spine density due to mTOR hyperactivation, reversible by RAPA.^48^ In contrast, our human phNPC model did not show or only partially showed rescue of pruning‐associated genes with RAPA, suggesting limited effectiveness of RAPA in normalizing synaptic development disruptions caused by *DEPDC5* mutations—at least in early DIFF stages as modeled here. Morphological data support this hypothesis. However, the clinical relevance to patients remains uncertain due to a lack of corresponding patient‐derived data. Furthermore, because the DIFF‐phNPC model corresponds to prenatal neuronal stages, its applicability to later developmental stages, such as adolescence when pruning primarily occurs, remains to be confirmed. Overall, we conclude that synaptic pruning mechanisms might be at the hallmark of DEPDC5 knockout, which cannot be targeted by RAPA treatment.

### 4.6 Role of p53 pathway

Notably, our analysis identified robust enrichment of p53‐related cell cycle mechanisms. The tumor suppressor p53 modulates responses to various cellular stresses, including DNA damage and oxidative stress. Feedback interactions between mTOR and p53 pathways have been associated previously with epileptic seizures.^49^ *MDM2*, a critical negative regulator of p53, was differentially expressed in both proliferation and differentiation conditions and normalized by RAPA treatment. Dysregulation of p53 and *MDM2* is a proposed mechanism in temporal lobe epilepsy pathology.^50^ Faster differentiation observed in DEPDC5 KO cells may result from these p53 pathway alterations.^51^ In addition, enrichment of the p53‐related transcription factor motif KLF4 in differentiated cells suggests that dysregulated p53 signaling significantly impacts neuronal differentiation and potentially links *DEPDC5* loss to epilepsy‐related mechanisms.^52^ The involvement of another p53‐interacting transcription factor, WT1, further supports this hypothesis. Finally, disruptions in KLF7, another identified TF here, have been linked to autism‐like phenotypes in animal models, reinforcing the potential broader impact of *DEPDC5*‐associated transcriptional changes.^53^

### 4.7 Limitations

The here implemented lentivirus KO‐strategy within bulk cultures induces a complete knockout and thus may represent only some of the patients, specifically those with mosaic double hits leading to KO of DEDPC5 on both alleles, but not patients carrying a heterozygous loss of DEPDC5 only. Thus, the study here allows us to draw conclusions about DEPDC5 loss and RAPA treatment effects at a general level only. Analysis of heterozygous phNPCS KO is limited because the here implemented primary cell system cannot be used for single‐clone expansion. Lentiviral transduction strategy leads to random but stable insertion of the gRNA/Cas9 cDNA sequences into the host genome and thus to a continuous activity of Cas9—as long as the gRNA can bind to the target sequence. The antibiotic selection of cells ascertained that only cells with integrated target sequences survive and thus we guarantee a full knockout. Although this approach might explain transcriptional difference between the KOs, our conservative analysis by only reporting replicated findings after correction for multiple testing ensures reliability of the findings here. At the protein level, we confirm this complete knockout of DEPDC5. The bulk‐culture approach here has the additional advantage that single clones are prone to the effects of individual random insertions, whereas in bulk cultures these effects will not be specific.

Our study is limited in that it can give an overall insight into the general transcriptomic signature only. Bulk RNA sequencing allows a general conclusion on the altered transcriptomics but does not allow analyses at the level of single cells or cellular subtype. In some patients the homozygous mosaic knockout could be cell type specific. Here, single‐iscell RNA sequencing would allow investigation of the effect of the KO by their respective transcriptomically inferred subtypes. Further studies should investigate the complex interaction of heterozygous, wild‐type, and KO cell types.

The RAPA rescue in proliferating cells only reflects an acute treatment, as the cells undergo continued proliferation in contrast to the differentiating cells; thus, comparison between the chronic treatments in DIFF cells is limited.

Finally, although our results contribute to understanding the potential involvement of mTOR‐independent pathways in DEPDC5 pathology, drawing direct conclusions about clinical outcomes is beyond the scope of this study.

## 5 CONCLUSION

We here propose a pathological mechanism involving alterations of p53, mediated by KLF transcription factor changes, yielding downstream mechanisms converging on the observed morphological and clinical phenotypes. We further identified that the gene expression changes can only be partly attributed to mTOR and specifically dissected that mTOR‐independent pathway changes share common biological functions such as pruning. We conclude that it is likely that presentation of ASD and epilepsy is a product of altered p53 mechanisms leading to altered neuronal development in the brain; however, the consequences thereof are not fully reversible by RAPA treatment. Thus, future studies need to investigate how potential mTOR‐independent changes can be targeted and repaired.

## AUTHOR CONTRIBUTIONS

Conceptualization, Methodology, Supervision, and Project Administration: A.G.C. and D.H. Investigation: M.J., J.L., D.H., and S.L. Software: A.G.C. and A.K. Resources: S.M., E.U., and C.M.F. Writing – Original Draft: M.J., A.G.C., D.H. Writing – Review & Editing: all authors. Funding Acquisition: F.R., K.M.K., and A.G.C. Visualization: R.W., A.G.C., D.H., and M.J.

## FUNDING INFORMATION

Goethe University, Frankfurt am Main, Germany, which is funded by The Hessian State Ministry for Higher Education, Research and the Arts (HMWK).

## CONFLICT OF INTEREST STATEMENT

The authors declare no conflict of interests interfering with any parts of this manuscript.

## ETHICS STATEMENT

Cells were kindly provided by Daniel Geschwind (UCLA, Los Angeles, CA, USA), and experiments were positively reviewed by the institutional review board at UCLA. We confirm that we have read the Journal's position on issues involved in ethical publication and affirm that this report is consistent with those guidelines.

## Supporting information


Appendix S1.

Table S1.

Table S2.

Table S3.

Table S4.

Table S5.

## Data Availability

RNA sequencing data are available as Gene Expression Omnibus resource: https://www.ncbi.nlm.nih.gov/geo/query/acc.cgi?acc=GSE240337 Repository: https://github.com/KJPMolgenLab/DEPDC5_D62_Analysis.git Markdowns: https://kjpmolgenlab.github.io/DEPDC5_D62_Analysis/
