# Supplementary material for: The transcriptomic signature of DEPDC5 KO induced mTOR hyperactivation in human neurons and its response to rapamycin treatment
Source: Epilepsia. 2025 Jul 24;66(11):4538–53. doi: 10.1111/epi.18549 (PMC12661282; doi:10.1111/epi.18549)
Supplement: Supplementary file 1 — Appendix S1. [file EPI-66-4538-s002.docx]

Supplementary information for

The transcriptomic signature of DEPDC5 KO induced mTOR-hyperactivation in human neurons and its response to rapamycin treatment

Mattson S. O. Jones^1,2^, Silvia Lindlar^1^, Johannes Ludwig^1^, Regina Waltes^1^, Afsheen Kumar ^1^, Sophie v. Brauchitsch^2,3^, Andrea Rossi^4^, Evelyn Ullrich^5,6,7^, Stefan Momma^8^, Christine M. Freitag^1^, Jasmin K. Hefendehl^9,10^, Karl Martin Klein^2,3,11^, Felix Rosenow^2,3^, Denise Haslinger*^1,2^ and Andreas G. Chiocchetti*^1,2^

^1^Autism Therapy and Research Center of Excellence, Department of Child and Adolescent Psychiatry, Psychosomatics and Psychotherapy, University Hospital, Goethe University Frankfurt, Frankfurt am Main, Germany

^2^Center for Personalized Translational Epilepsy Research (CePTER), Goethe University Frankfurt, Frankfurt am Main, Germany

^3^Epilepsy Center Frankfurt Rhine-Main, Center of Neurology and Neurosurgery, Goethe-University Frankfurt, Schleusenweg 2-16 (Haus 95), 60528, Frankfurt am Main, Germany

^4^IUF-Leibniz Research Institute for Environmental Medicine, 40225 Düsseldorf, Germany

^5^Experimental Immunology, Children's University Hospital, Goethe University Frankfurt, Frankfurt am Main, Germany

^6^Frankfurt Cancer Institute (FCI), Goethe University Frankfurt, Frankfurt am Main, Germany

^7^ German Cancer Consortium (DKTK), Partner Site Frankfurt/Mainz, Frankfurt am Main, Germany

^8^Institute of Neurology (Edinger Institute), Goethe University, Frankfurt am Main, Germany

^9^Institute of Cell Biology and Neuroscience, University of Frankfurt, D-60438 Frankfurt am Main, Germany

^10^Neurovascular Disorders, Buchmann Institute for Molecular Life Sciences, University of Frankfurt, D-60438 Frankfurt am Main, Germany

^11^Departments of Clinical Neurosciences, Medical Genetics, and Community Health Sciences, Hotchkiss Brain Institute & Alberta Children's Hospital Research Institute, Cumming School of Medicine, University of Calgary, Calgary, Alberta, Canada

* Authors contributed equally (shared last)

Corresponding author: Andreas G. Chiocchetti, [andreas.chiocchetti@med.uni-frankfurt.de](mailto:andreas.chiocchetti@med.uni-frankfurt.de)

## Table of Content

[Table of Content 2](#_Toc193872311)

[Supplementary Methods 4](#_Toc193872312)

[Experimental model and subject details 4](#_Toc193872313)

[Cell line generation and cultivation 4](#_Toc193872314)

[Cell line differentiation 4](#_Toc193872315)

[Generation of DEPDC5 KO in phNPCs 5](#_Toc193872316)

[sgRNA design 5](#_Toc193872317)

[LentiCRISPRv2 cloning 5](#_Toc193872318)

[Lentivirus Production 6](#_Toc193872319)

[Transduction 6](#_Toc193872320)

[Confirmation of DEPDC5 KO in phNPCs 7](#_Toc193872321)

[Sanger sequencing 7](#_Toc193872322)

[Western blot 7](#_Toc193872323)

[Functional characterization of DEPDC5 KO vs NTC 8](#_Toc193872324)

[Immunocytochemistry 8](#_Toc193872325)

[Starvation Assay (mTOR hyperactivation experiments) via Western blotting 9](#_Toc193872326)

[Transcriptomic analysis (3’-mRNA sequencing) 9](#_Toc193872327)

[Morphology and cell counting 10](#_Toc193872328)

[Quantification and statistical analysis 10](#_Toc193872329)

[RNA-Sequencing Analysis 10](#_Toc193872330)

[Weighted gene co-expression network analysis (WGCNA) 11](#_Toc193872331)

[Gene List enrichment testing 11](#_Toc193872332)

[Protein-Protein interaction analysis 11](#_Toc193872333)

[MApping the Genetics of neuropsychological traits to the molecular NETwork (MAGNET) 12](#_Toc193872334)

[Statistical analysis of Morphology 12](#_Toc193872335)

[Data 12](#_Toc193872336)

[Code 12](#_Toc193872337)

[Information on reagents, resources on suppliers and catalog numbers 13](#_Toc193872338)

[Supplementary Material 16](#_Toc193872339)

[Supplementary Tables 16](#_Toc193872340)

[Supplementary Table S1: Genelists 16](#_Toc193872341)

[Supplementary Table S2: WGCNA association 16](#_Toc193872342)

[Supplementary Table S3: GOres 16](#_Toc193872343)

[Supplementary Table S4: DEX Genelists 16](#_Toc193872344)

[Supplementary Table S5: All genes 16](#_Toc193872345)

[Supplementary Figures 17](#_Toc193872346)

[Supplementary Figure S1: 17](#_Toc193872347)

[Supplementary Figure S2: 18](#_Toc193872348)

[Supplementary Figure S3: 19](#_Toc193872349)

[Supplementary Figure S4: 20](#_Toc193872350)

[Supplementary Figure S5: 20](#_Toc193872351)

[Supplementary Figure S6: 22](#_Toc193872352)

[References 22](#_Toc193872353)

# Supplementary Methods

## Experimental model and subject details

We utilized primary human neural progenitor cells (phNPCs; D62) extracted from the developing neocortex at gestational week 14 from previously established biobanks and protocols (1–5). The cells were kindly provided by Daniel Geschwind (UCLA, Los Angeles USA), and experiments were positively reviewed by the IRB ethics board at UCLA.

## Cell line generation and cultivation

The cells were isolated and grown as neurospheres and thereafter transferred to cell culture plates and grown as 2D culture (2). For cell maintenance and proliferation, culture plates were coated with 5µg/mL poly-L-ornithine (PO; 0.01% solution; Sigma) diluted in Dulbecco's phosphate-buffered saline (DPBS; Gibco) and incubated for 1-2 hours at 37°C. The solution was aspirated, wells washed once with DPBS and re-coated with 5 µg/mL fibronectin (F; Sigma) in DPBS over night (ON) at 37°C. Fibronectin solution was aspirated and plates dried under a sterile cell culture hood for 3 hours. Dried PO-F plates were wrapped in parafilm (Bemis Company Inc.) and stored at 4°C for no more than 1 month. phNPCs were expanded on PO-F coated cell culture plates and maintained in proliferation media consisting of Neurobasal A w/o Glutamine (Gibco) supplemented with 1X Antimycotic-Antibiotic (Sigma), 1X GlutaMAX (100X, Gibco), 10% Knockout serum replacement (Gibco), 1µg/mL Heparin (AppliChem), 20pg/µL EGF (Peprotech) and 20pg/µL FGF (Peprotech). Medium was changed every 2-3 days by refreshing half of the total volume supplemented with the double amount of epidermal growth factor (EGF) and fibroblast growth factor (FGF; final concentrations: 20pg/µl). phNPCs reaching 80-90% confluency were split 1:2 by first washing with DPBS and then detached with Accutase (Sigma) by incubating for 1-2 minutes (min) at 37°C. Afterwards, the cells were fully detached with in Proliferation medium and then collected and centrifuged at 300xg for 3 min. The cell pellet was resuspended in fresh Proliferation medium supplied with EGF/FGF. phNPCs were always kept at a confluency higher than 50% as lower confluency significantly slowed growth rates and isolated cells separated from colonies spontaneously differentiate or die.

## Cell line differentiation

For neural differentiation, the phNPCs were cultured on coverslips coated with poly-L-ornithine (PO) and laminin (L; Corning). phNPCs were differentiated in Neurobasal A w/o Glutamine (Gibco) with 1X Antibiotic-Antimycotic (Sigma), 1X B27 serum supplement with Vitamin A (50X; Gibco), 1X GlutaMAX, 20μM Bucladesine (Caymen Chemical), 10mM Potassium Chloride (KCl), 500ng/mL Retinoic Acid (RA; Sigma), 10pg/μL BDNF (Immunotools), and 10pg/μL NT-3 (Immunotools). Cells were differentiated for 4 weeks with 2-3 half media changes per week with doubling of Bucladesine, RA, BDNF and NT-3 to achieve the above-mentioned final concentrations.

## Generation of DEPDC5 KO in phNPCs

As D62 cell clones cannot be expanded from single cells, all experiments were performed on a mixed KO cell population. In addition, D62 exhibited low transfection efficiencies and thus a lentiviral CRISPR/Cas9 strategy was adapted.

### sgRNA design

The CRISPR-Cas9 technique was used to induce mutations leading to premature stop codons (6). We here designed sgRNAs targeting the area of a patient-specific mutation site: The mutation (c.21C>G) changes a tyrosine in exon 2 of *DEPDC5* and was identified in an Australian family exhibiting ASD, epilepsy or both (7). Exon 2 was shown to be present in all isoforms of *DEPDC5* (Ensembl Genome Browser www.ensembl.org). It should be mentioned that *DEPDC5* exon 1 is a pseudo exon and sometimes exon 2 is referred to as exon 1. However, for consistency with other literature we chose to adhere with exon 2. Two *DEPDC5*-Ex2 targeting sgRNAs (*DEPDC5*_Ex2.1/Ex2.2) and one non-targeting control (NTC) were designed using a CRISPR design tool (currently discontinued; <https://zlab.bio/guide-design-resources>). sgRNA targets were verified for minimal off-target effects, lack of neuronal related genes and lack of overlapping exon regions using the CRISPR-Cas9 online *predictor* CCTop (cctop.cos.uni-heidelberg.de:8043; 8).

It has been reported that transfection methods using phNPCs result in relatively low transfection rates (5-20%) and tend to be quite variable depending on method, age of fetal material or the length of time the cells are in vitro (9, 10). Preliminary experiments yielded very low transfection efficiencies (~10%; data not shown) therefore lentivirus was used adapting the widely published lentiCRISPRv2 plasmid (Plasmid #52961; Addgene; 6). sgRNA oligomers were designed as described above and a guanine was added at the 5‟ end to increase U6 promotor efficiency (<https://www.addgene.org/crispr/zhang/>).

### LentiCRISPRv2 cloning

Ordered oligos (Sigma) were phosphorylated and annealed using 10μM forward and reverse primers, 1X T4 PNK ligation buffer (NEB: New England Biologicals), 1mM ATP (NEB) and 0.5U/μL T4 PNK (NEB). After annealing at 37°C for 1hr, 95°C for 5 min and ramping down to 25°C at 5°C/min, resulting oligo duplexes were diluted 1:250. sgRNAs were cloned into plasmids using 5ng/μL lentiCRISPRv2, 4nM annealed oligo duplex, 1x FastDigest Buffer (Fermentas), 10mM DTT (Applichem), 10mM ATP (NEB), 0.05 U/µL FastDigest BsmBI and 0.5U/μL T4 DNA ligase (Thermo). The ligation reaction was conducted in a thermocycler (SensoQuest Labcycler) 37°C for 5 min and 23°C for 5 min for 6 cycles and stored at 4°C. Unwanted recombination products were removed using 11μL ligation reaction, 1x PlasmidSafe buffer (1X), 10mM ATP (1mM) and 0.6 U/μL PlasmidSafe exonuclease (Lucigen) at 37°C for 30 min.

Plasmids were transformed into 50μL Stbl3 E. coli cells (NEB) by heat shock. After 30 min on ice, the cell/plasmid mix was transformed for 30 s at 42°C and cooled for 5 min on ice. 950μL prewarmed NEB 10-beta/Stable Outgrowth Medium without antibiotics was added and cells were incubated at 30°C at 225rpm for 1h. Transformed Stbl3 bacteria were placed onto warmed LB agar plates (30°C) with 100µg/mL ampicillin and incubated overnight at 37°C. Individual colonies were picked and expanded in LB medium with 100µg/mL ampicillin for 24 hours, plasmids were extracted (Nucleobond Xtra Midi Kit; Macherey-Nagel) and validated with Sanger-sequencing (Eurofins) using forward primers targeting the U6 promotor. Successful insertion of sgRNAs into lentiCRISPRv2 resulted in the constructs NTC (lentiCRISPRv2_NTC), Dep2.1 (lentiCRISPRv2_Dep2.1) and Dep2.2 (lentiCRISPRv2_Dep2.2).

### Lentivirus Production

For lentiviral production, HEK293T cells (ATCC) were expanded in 10% Fetal Bovine Serum (Gibco), 1mM Sodium Pyruvate (100mM; Gibco), 1X Pen/Strep (10,000 units/mL Penicillin, 10,000 μg/mL Streptomycin; Gibco) in Dulbecco's Modified Eagle Medium (DMEM with Glutamine, 25mM HEPES, 4.5g/L Glucose; Gibco). Subsequently, cells were transfected by calcium chloride transfection composed of 2X Hank’s Balanced Salt Solution (Bioworld), 250mM CaCl2 (VWR International) and 25µM Chloroquine (Bioworld) using transfer plasmids (lentiCRISPRv2 with the respective inserts) and helper plasmids: pMDLg/pRRE (Plasmid: #12251; Addgene), pRSV/Rev (Plasmid: #12253; Addgene) and pMD2.G (#12259; Addgene). Cell culture media containing viral particles were subsequently collected every 24 hours for three days and concentrated via ultracentrifugation at 50,000xg for 1 hour at 4°C (Avanti JXN-30; Beckman Coulter) three days after transfection. Viral titer was calculated by qPCR Titration Kit according to manufacturer’s instructions (abm).

### Transduction

Utilization of polybrene to enhance transduction resulted in cell death and therefore the cells were transduced with pure virus. Lentiviral integrations follow a Poisson distribution of randomly occurring independent events where the numbers of infected cells are predicted to have a single integration at 30% (11). Therefore, we aimed for a transduction efficiency of 30% to ensure single integrations and to reduce the likelihood of double integrations. D62 cells were transduced with lentiCRISPRv2 constructs at MOI 3.3 (containing the sequence for Cas9 as well as the sgRNAs for NTC, Dep2.1 and Dep2.2, respectively) for 18 hours, followed by full media changes. After another 72 hours, transduced cells were selected with 1μg/mL puromycin (Sigma) for 5-7 days. Surviving cells were then pooled together across multiple dishes for each resulting cell line to ensure confluency above 70% following selection resulting in bulk cultures.

## Confirmation of DEPDC5 KO in phNPCs

### Sanger sequencing

To confirm *DEPDC5* KO on DNA level we performed Sanger Sequencing. DNA was extracted from cell pellets using the MasterPure DNA Purification Kit (Lucigen) following the manufacturer’s protocol. Extracted DNA was resuspended in TE Buffer and concentration measured using a nanophotometer (Implen). Primers were designed using the IDT PrimerQuest Tool (https://eu.idtdna.com/pages/tools/primerquest). The specificity was rechecked using the National Center for Biotechnology Information (NCBI) nucleotide Basic Local Alignment Search Tool (BLAST) (https://blast.ncbi.nlm.nih.gov/Blast.cgi). Confirmation of DNA sequences for CRISPR-Cas9 *DEPDC5* KOs utilized the generation of polymerase chain reaction (PCR) fragments from exon 2 validation primers and Sanger sequencing (Eurofins). Plasmid ligation constructs were directly sequenced from the plasmid using the U6 promotor. For PCR, 20ng DNA was mixed with 200nM forward and reverse primer pairs, 1X Biozym Taq Polymerase (Biozym) and 200μM dNTPs (Biozym). After an initial denaturation step of 95°C for 1 minute, DNA was amplified at 95°C for 15 seconds (s), 60°C for 15 s and 72°C for 30 s for a total of 40 reaction cycles in a thermocycler (SensoQuest Labcycler). Amplified and purified PCR fragments were sent for Sanger sequencing (Eurofins) using forward or reverse primers designed outside sgRNA target regions for *DEPDC5* exon 2. Plasmids were sequenced with the U6 forward primer. Resulting ab1 files were analyzed using Benchling (Benchling.com).

### Western blot

The loss of function of the DEPDC5 protein by absent protein expression was confirmed by Western blot. A total of 2-3x10^6^ poliferating cells were lysed with modified RIPA buffer containing 50mM Tris/HCl, pH8, 0.1% NP-40, 0.5% Na-deoxycholate, 150mM NaCl, 2mM MgCl2, 0.1% SDS, 40μL/mL Proteinase Inhibitor Cocktail, 1mM Sodium Orthovanadate, 100mM NaF, 2mM DTT (all Sigma), 1:1000 Pierce Universal Nuclease (Thermo Fisher). Cells were vortexed every 5 min for 30 min on ice and subsequently centrifuged for 30 min at 17,000xg at 4ºC. Lysate was collected and concentrations were measured using Pierce BCA Protein Assay Kit (Life Technologies) according to manufacturer’s recommendation; measurements were done on a Multiskan FC 96 well nanophotometer (Implen) at 570nm. 50μg of protein was loaded onto a 10% SDS gel [resolving gel: 10% acrylamide/bis-acrylamide (37.5:1; Serva), 0.375M Tris pH 8.8, 0.1% SDS (both AppliChem), 0.05% APS, 0.1% TEMED (both Carl Roth); stacking gel: 4% acrylamide/bis-acrylamide (37.5:1; Serva), 0.125M tris pH8.8, 0.1% SDS (both AppliChem), 0.1% APS, 0.2% TEMED (both Carl Roth)]. To collect samples in the stacking gel, the gels were run at 50V for 15 min and at 150V until complete separation in 1X running buffer (25mM Tris, 192mM glycine, 0.1% SDS (AppliChem).

Gels were semi-dry blotted (Trans-Blot Semi-Dry Transfer Cell; Bio-rad) on a polyvinylidene difluoride (PVDF) membrane (Immobilon-FL) for 80 min at 1mA/cm2in Blotting Buffer (48mM Tris, 39mM glycine, 20% methanol). Blotted membranes and transferred gels were subjected to Ponceau S-Solution (AppliChem) or PageBlue Staining Solution (Thermo Scientific) respectively and visualized using FluorChem Q Imaging System (ProteinSimple). Ponceau solution was removed with 3 washes of 1x PBS (Thermo Fisher) + 0.1% Tween-20 (PBST; AppliChem;) and blots were incubated for 1 hour in blocking buffer (iBind Flex Solution; Invitrogen) on an orbital shaker at RT. Blots were then incubated with primary antibody (diluted 1:700 Oct4, Pax6, Sox2; 1:5000 GAPDH; 1:1000 for the others) in blocking solution overnight at 4ºC followed by 3 washes at 3 min with 1x PBS + 0.1% Tween-20 (PBST). Blots were then incubated with secondary antibodies (1:5000) coupled to horseradish peroxidase for 1 hour at RT followed by 3 washes. Visualization was performed using a 30 s incubation of Trident Femto Western Blot ECL Solution (Genetex) yielding a chemiluminescent signal and images were taken using a FluorChem Q Imaging System (Protein Simple) after 1, 5 and 10 min. Blots were then stripped between stains using 100mM 2-mercaptoethanol (Carl Roth), 2% SDS, 62.5mM tris pH 6.7 (AppliChem) for 30 min at 50ºC on a roller, washed 3 times with PBST, and reblocked and immunostained as described above.

## Functional characterization of DEPDC5 KO vs NTC

### Immunocytochemistry

For immunocytochemistry, phNPCs were seeded on Polyornithin-Fibronectin (PO-F) (Proliferation) or PO-Laminin (PO-L) (Differentiation) coated coverslips with a density of 2.5x10^4^cells/cm^2^ and incubated in proliferation or differentiation media respectively. For rescue experiments, DEPDC5-KO cells were treated with 100nM RAPA (Absource Diagnostics); 18h for proliferation and over the time course of 1 month for differentiation. PO-L coverslips were prepared by sterilization for 48 hours in a 93% nitric acid solution (Avantor) at room temperature (RT) on an orbital shaker. Nitric acid was removed followed by three washes in ddH20 and air dried after washing with 100% ethanol overnight (ON) under a sterile workbench. Nitric acid-washed coverslips were collected and stored at RT until further use. Acid-washed coverslips were precoated with 5μg/mL PO in DPBS for a minimum of 1 hour at 37°C. After aspiration and DPBS washing, the coverslips were then coated with 10μg/mL laminin in DPBS and incubated overnight at 37°C. PO-L coated coverslips were then immediately used or stored at 4°C in PBS for no more than 1 week.

hNPCs and neurons were visualized with respective markers. For this, cells were fixed in 4% paraformaldehyde + 4% sucrose (Electron Microscopy Sciences; Sigma) in 1X PBS with calcium and magnesium for 15 min at room temperature (RT). Fixed cells were then washed 3 times and submerged in 1X PBS until staining. Stains were performed by permeabilization with 0.1% Triton X-100 in PBS for 15 min at RT followed by immediate blocking in 10% goat and donkey serum (Merck) in 1X PBS+0.02% Tween-20 (PBST) for 1 hour at RT. Fixed cells were then submerged in 20μL diluted primary antibodies in antibody solution (1:500 dilution in 5% goat and donkey serum in 1X PBST; overnight in a humidified chamber at 4°C followed by 3 washes with PBST and subsequent 1:1000 secondary antibody incubation in antibody solution for 1 hour at RT. Stained cells were washed a final 3 times in 1X PBST, washed one time in double-distilled H_2_O and mounted to ethanol-washed microscope slides with Prolong Gold Antifade with DAPI (Thermo Fisher). Cells were visualized with a Nikon Eclipse Ti Confocal Microscope equipped with a spinning disc unit (CSUW1, Andor) and LED lasers at wavelengths 405, 488, 561 and 639. Visualization was done with Nikon Elements software (version 4.60.00 Build 1171).

### Starvation Assay (mTOR hyperactivation experiments) via Western blotting

2-3x10^6^ proliferating cells were incubated in 10cm cell culture dishes for 90 min in Neurobasal A or Neurobasal A without amino acids (US Biological) supplemented with 3.7g/L Sodium Bicarbonate (Gibco). For rescue experiments, cells were incubated with 100nM RAPA (Absource Diagnostics). Immediately following incubation, cells were collected in ice cold DPBS, centrifuged at 500xg and immediately lysed in RIPA buffer (Western blotting above). Subsequently, protein extracts underwent Western Blotting as described above.

### Transcriptomic analysis (3’-mRNA sequencing)

To observe the effects of *DEPDC5* KO on the transcriptome, 3’-mRNA sequencing analysis was performed. RNA was extracted using the Nucleospin RNA Kit (Macherey-Nagel) from three biological replicates of NTC, Dep2.1, and Dep2.2 with or without 100nM RAPA for progenitors (18h of RAPA on proliferating cells) and after 1 month of neuronal differentiation. Steps were followed per manufacturer’s instructions and RNA was collected in RNase-free H2O. RNA concentrations were measured using a nanophotometer (Implen). All samples passed quality check using a Tapestation 4200 (Agilent Technologies) with an RNA integrity number (RIN) >7. RNA libraries were generated with Quantseq 3’-mRNA Library Prep (Lexogen) and sequenced on a HiSeq 2500 V4 (High Output Mode) with a coverage of 10M Raw Reads per sample and a standard read length of 1x50bp (NGS Core Facility Bonn). Raw reads were quality controlled (FastQC; https://www.bioinformatics.babraham.ac.uk/projects/fastqc/), trimmed (trimmomatic; 12) and aligned to hg38 (Rsubread; 13) using the standard settings as provided in the manual. Feature counts were extracted and used for subsequent statistical analysis. All samples passed quality control. For statistical analysis see below.

### Morphology and cell counting

Analysis of cell sizes as well as neuronal, astrocyte and phNPC counts were done using FIJI ImageJ software (v. 1.53f51). Sholl analysis of MAP-stained cells was performed with the neuroanatomy plugin Simple Neurite Tracer (version 4.0.3) with a radius step of 1, semi-log method and “Best fitting‟ degree settings. Dendrite lengths were collected from Sholl analysis measurements. Twenty cells were counted per cell line and per condition.

## Quantification and statistical analysis

### RNA-Sequencing Analysis

Full code and results of RNA Sequencing data analysis is available online as R-markdown output <https://kjpmolgenlab.github.io/CePTER_RNASeq/index.html>.

Count matrices were based on entrez gene ids and checked for non-unique annotations. Non-unique annotations were merged by sum. Visual inspection of count distributions per sample and per gene as well as clustering analysis of raw and normalized counts per million reads (cpm) were performed to check for technical outliers. We removed genes with no variance across samples or which were detected in less than 50% of the samples. After cleaning, the average read per sample was 7,195,819 (SD 345,374). In total 14,664 genes were removed due to low reads, resulting in 13,731 genes passing quality criteria. Count normalization and the statistical analysis was performed on cleaned data using DESeq2 with the full model being:

cpm ~ intercept + factor(gRNA) + factor (DIFF) + factor (RAPA) + factor (Cell line)

To confirm replication of technical and biological replicates, hierarchical clustering (hclust) analysis was performed on the top 2000 genes by variance. The replicates were inspected based on the Euclidean distance, principal component analysis (GLM-PCA), and multidimensional scaling (MDS).

For group comparison and identification of differentially expressed genes (DEX), raw counts were loaded into DESeq2 using the “DESeqDataSetFromMatrix” function. Differential expression was estimated using the function “DESeq” with standard options. P-values were false discovery rate (fdr) corrected for the number of genes tested. Genes were considered to be significant if the fdr was < 0.05 in both KO cell lines compared to the NTC control, respectively. Resulting deregulated gene datasets were analyzed based upon the KEGG Mapper Tool (https://www.genome.jp/kegg/mapper/search.html), Entrez gene database summary https://www.ncbi.nlm.nih.gov/gene, or Genecard database summary (https://www.genecards.org/) and clustered to known pathway or functional mechanisms related to the mTOR pathway. Isolated genes were further investigated for ASD and epilepsy disease gene overlap.

### Weighted gene co-expression network analysis (WGCNA)

Normalized count data was variance stabilized (getVarianceStabilizedData) and log2 transformed to model normal distribution (14). Best softPower threshold for calculating the adjacency matrix was estimated based on the Scale free topological model fit (R² > 0.8) and the Mean connectivity (scree plot criterion). softPower was set to 6. We calculated the unsigned topological overlap matrix and calculated modules based on the fast clust (hierarchical clustering) dendrograms implementing the cutreeDynamic including the option that the PAM algorithm respects dendrogram structure in the identification of gene-sets. The minimum number of genes within a set was defined as 50. Module Eigengenes (first principal component) were calculated. Modules were merged if they clustered together with a distance <0.2 (mergeCloseModules). Differences between KO and NTC were tested for each condition with each cell lineseparately implementing linear modeling as described above correcting p-values for the number of modules tested.

### Gene List enrichment testing

To test whether DEPDC5 KO is associated with the disease of interest, the differentially expressed genes were tested for enrichment using fisher exact test in published data sets of high-risk genes for epilepsy (Epi25 https://epi25.broadinstitute.org/; 15), Autism Spectrum Disorders (https://gene.sfari.org/, 16–18), Fragile X Syndrome (19), Tuberous Sclerosis (20, 21), Intellectual Disability (22, 23), or Schizophrenia (24, 25); see Table S2). Gene lists were considered as associated with DEPDC5-KO if the odds ratio (OR) was greater 1 and the Bonferroni adjusted p-value < 0.05 (Fisher exact test). GO term enrichment analysis was performed implementing the gprofiler2 (26) package. We used the correction_method=”g_SCS'' to account for multiple testing as well as the diacyclic graph structure of the ontologies. All genes passing quality control in our analysis were set as a reference gene-universe.

### Protein-Protein interaction analysis

In addition we performed STRING_DB protein-protein interaction analysis using the Cytyoscape (V 3.10.2) STRING plugin. Confidence threshold cutoff was set to 0.4 and an a maximum of 100 interaction partners were added. Retrieved networks were clustered (MCL algorithm) with an inflation value of 4. Enrichment testing was performed using the innate Enrichment function of the STRING-App in cytoscape for the resulting clusters with more than 5 nodes.

### MApping the Genetics of neuropsychological traits to the molecular NETwork (MAGNET)

To evaluate developmental age and brain region of the genes differentially expressed due to DEPDC5 KO, transcriptomic data was integrated into the MApping the Genetics of neuropsychiatric traits to the molecular NETworks of human brain (MAGNET) pipeline (https://molgenlab.shinyapps.io/MAGNET_lite_V2/; 27). This in-house pipeline was generated using the Allen Brain Atlas (28) using 1,340 tissue samples taken from one or both hemispheres of 57 postmortem brains varying over age. Transcripts were tested with Fisher’s exact test for significant enrichment in the 29 lists of genes corresponding to co-regulated modules activated during brain development, as published in the original publication (adjusted p-value<0.05).

### Statistical analysis of Morphology

Group differences between morphological analysis were tested using two-sample unequal variance T-test where significance was defined if p < 0.05. All samples were compared to their respective controls (NTC vs Dep2.1 or Dep2.2). Mean measurements and standard error of the mean were calculated for each morphological parameter.

## Data

<https://www.ncbi.nlm.nih.gov/geo/query/acc.cgi?acc=GSE240337>
Access token for reviewers: **sryzeiyqrzavhkx**

## Code

**Repository**[**https://github.com/KJPMolgenLab/DEPDC5_D62_Analysis.git**](https://github.com/KJPMolgenLab/DEPDC5_D62_Analysis.git)

**Markdowns**[**https://kjpmolgenlab.github.io/DEPDC5_D62_Analysis/**](https://kjpmolgenlab.github.io/DEPDC5_D62_Analysis/)

### Information on reagents, resources on suppliers and catalog numbers

| **REAGENT or RESOURCE** | **SOURCE** | **IDENTIFIER** |
| --- | --- | --- |
|  | | |
| **Antibodies** | | |
| Mouse anti-Beta Tubulin 3 (Tuj1) | Genetex | Cat# GTX130245; RRID: AB_2886220 |
| Guinea Pig anti-GFAP | Synaptic systems | Cat#173 004; RRID: AB_10641162 |
| Mouse anti-MAP2 | Millipore | Cat# MAB3418; RRID: AB_11212326 |
| Mouse anit-Ki67 | BD Transduction Laboratories | Cat#610968 |
| Goat anti-mouse (conjugate 488) | Thermo Fisher | Cat# 35502; RRID: AB_844397 |
| Donkey anti-guinea pig (conjugate Cy3) | Millipore | Cat# AP193C; RRID: AB_92669 |
| Donkey anti-rabbit (conjugate 647) | Thermo Fisher | Cat# 31573; RRID: AB_2536183 (Discontinued) |
| Rabbit anti-AKT (pan) | Cell Signaling | Cat# 4691S; RIDD: AB_915783 |
| Rabbit anti-DEPDC5 | Abcam | Cat# ab185565; RRID: NA |
| Mouse anti-GAPDH | Santa Cruz | Cat# sc-166545; RRID: AB_2107299 |
| Rabbit anti-Oct4 | Genetex | Cat# GTX101497; RRID: AB_10618784 |
| Rabbit anti-Pax6 | Genetex | Cat# GTX113241; RRID: AB_1951119 |
| Rabbit anti-phospho-S6 (Ser240/244) | Cell Signaling | Cat# 5364S; RRID: AB_10694233 |
| Rabbit anti-Phospho-AKT (Ser473) | Cell Signaling | Cat# 4060S; RRID: AB_2315049 |
| Rabbit anti-S6 ribosomal protein | Cell Signaling | Cat# 2217S; RRID: AB_331355 |
| Mouse anti-Sox2 | Genetex | Cat# GTX627404; RRID: AB_11162865 |
| anti-Nestin | Genetex | Cat# GTX116066; RRID: |
| Goat anti-mouse Horseradish peroxidase | Cell Signaling | Cat#7076; RRID: AB_330924 |
| Goat anti-rabbit Horseradish peroxidase | Cell Signaling | Cat#7074; RRID: AB_2099233 |
|  |  |  |
| **Chemicals, peptides, and recombinant proteins** | | |
| Serum Goat | Merck | Cat# 36601423; RRID: NA |
| Serum Donkey | Merck | Cat# 36601424; RRID: NA |
| Poly-L-ornithine | Sigma | Cat# P4957; CAS: 27378-49-0 |
| Fibronectin | Sigma | Cat# F1141; EC: 289-149-2 |
| Neurobasal A w/o phenol red | Gibco | Cat# 12349-015 |
| GlutaMAX | Gibco | Cat# 35050-038 |
| Antimycotic-Antibiotic | Sigma | Cat# A5955; CAS: 57-92-1 CAS 1397-89-3 CAS 113-98-4 |
| Knockout serum replacement | Gibco | Cat# 10828010 |
| Heparin | AppliChem | Cat# ; CAS: 9005-49-6 |
| EGF (Epidermal Growth Factor) | Peprotech | Cat# AF-100-15;  CAS: 62253-63-8 |
| FGF (Fiboblast Growth Factor) | Peprotech | Cat# 100-18B; CAS: 62031-54-3 |
| DPBS | Gibco | Cat# 14190250 |
| Accutase | Sigma | Cat# A6964 |
| Laminin mouse | Corning | Cat# 354232; CAS: 114956-81-9 |
| B27 serum supplement with Vitamin A | Gibco | Cat# 17504044 |
| Bucladesine | Caymen Chemical | Cat# 14408; CAS: 362-74-3 |
| Retinoic acid | Sigma | Cat# R2625; CAS: 302-79-4 |
| rh BDNF | Immunotools | Cat# 11343373; CAS: 218441-99-7 |
| rh NT-3 | Immunotools | Cat# 11343333 |
| Blasticidin | InvivoGen | Cat# ANT-BL-05; CAS: 2079-00-7 |
| Rapamycin | Absource Diagnostics | Cat# S1039 |
|  |  |  |
| **Critical commercial assays** | | |
| RevertAid H Minus First Strand cDNA Synthesis Kit | Thermo Fisher | Cat# 1632 |
| Nucleospin RNA kit | Macherey-Nagel | Cat# 740955.50 |
| NucleoSpin RNA/Protein, Mini kit for RNA and protein purification | Macherey-Nagel | Cat# 740933.10 |
|  |  |  |
| **Deposited data** | | |
| RNA sequencing  <https://www.ncbi.nlm.nih.gov/geo/query/acc.cgi?acc=GSE240337> | This paper | N/A |
| Code repository <https://github.com/KJPMolgenLab/DEPDC5_D62_Analysis.git> | This paper | N/A |
| Code markdowns  <https://kjpmolgenlab.github.io/DEPDC5_D62_Analysis/> | This paper | N/A |
|  |  |  |
|  |  |  |
| **Experimental models: Cell lines** | | |
| phNPCs from developing neocortex at gestational week 14, male, CEU | This manuscript; Geschwind lab, UCLA; | N/A |
| *DEPDC5* KO loss of function phNPCs | This manuscript | N/A |
| CB153 | Stefan Momma | N/A |
| HEK293T |  |  |
|  |  |  |
| **Oligonucleotides** | | |
| CRISPR sgRNA DEPDC5_Ex2.1 forward  CACCGCAAACTCGTCATCCACAAGA | This paper | N/A |
| CRISPR sgRNA DEPDC5_Ex2.1 reverse  AAACTCTTGTGGATGACGAGTTTGC | This paper | N/A |
| CRISPR sgRNA DEPDC5_Ex2.2 forward  CACCGTGCAAGATGAGAACAACAA | This paper | N/A |
| CRISPR sgRNA DEPDC5_Ex2.2 reverse  AAACTTGTTGTTCTCATCTTGCAC | This paper | N/A |
| CRISPR sgRNA NTC forward  CACCGTTCCGGGCTAACAAGTCCT | This paper | N/A |
| CRISPR sgRNA NTC reverse  AAACAGGACTTGTTAGCCCGGAAC | This paper | N/A |
| CRISPR validation primer (plasmid) Human U6 forward  GAGGGCCTATTTCCCATGATTCC | This paper | N/A |
| CRISPR validation primer (DEPDC5 exon 2) forward  CCCTTAGTTCCTGGATTCTGTG | This paper | N/A |
| CRISPR validation primer (DEPDC5 exon 2) reverse  TAGTCTGTTTAGTCGCCTGTTTAG | This paper | N/A |
|  |  |  |
| **Recombinant DNA** | | |
| Plasmid lentiCRISPRv2 | Addgene | Addgene Plasmid #52961 |
| pMDLg/pRRE | Addgene | Addgene Plasmid #12251 |
| pRSV/Rev | Addgene | Addgene Plasmid #12253 |
| pMD2.G | Addgene | Addgene Plasmid #12259 |
| Plasmid lentiCRISPRv2_NTC | This paper | N/A |
| Plasmid lentiCRISPRv2_Dep2.1 | This paper | N/A |
| Plasmid lentiCRISPRv2_Dep2.2 | This Paper | N/A |
|  |  |  |
| **Software and algorithms** | | |
| Nikon elements software (version 4.60.00 Build 1171) | Nikon | N/A |
| StepOne Plus software (version 2.3) | Thermo Fisher | N/A |
| FIJI ImageJ software (V. 1.53f51) | Schindelin et al. 2012 | <https://imagej.net/software/fiji/> |
| Neuroanatomy plugin Simple Neurite Tracer | Longair et al. 2011 | <https://imagej.net/plugins/snt/> |

# Supplementary Material

## Supplementary Tables

Supplementary table are provided as separate XLSX files

### Supplementary Table S1: Genelists

Published genelists used for enrichment analysis for the detected 13731 genes

### Supplementary Table S2: WGCNA association

Linear models of WGCNA Module Eigen-gene expression predicted by KO

### Supplementary Table S3: GOres

Gene enrichment analysis

### Supplementary Table S4: DEX Genelists

Replicated and significant (fdr <0.05) differentially expressed genes (DEX) across conditions

### Supplementary Table S5: All genes

Foldchanges and adjusted p-values for the comparison NTC versus DEPDC5KOs for the detected 13731 genes

# Supplementary Figures

## Supplementary Figure S1:


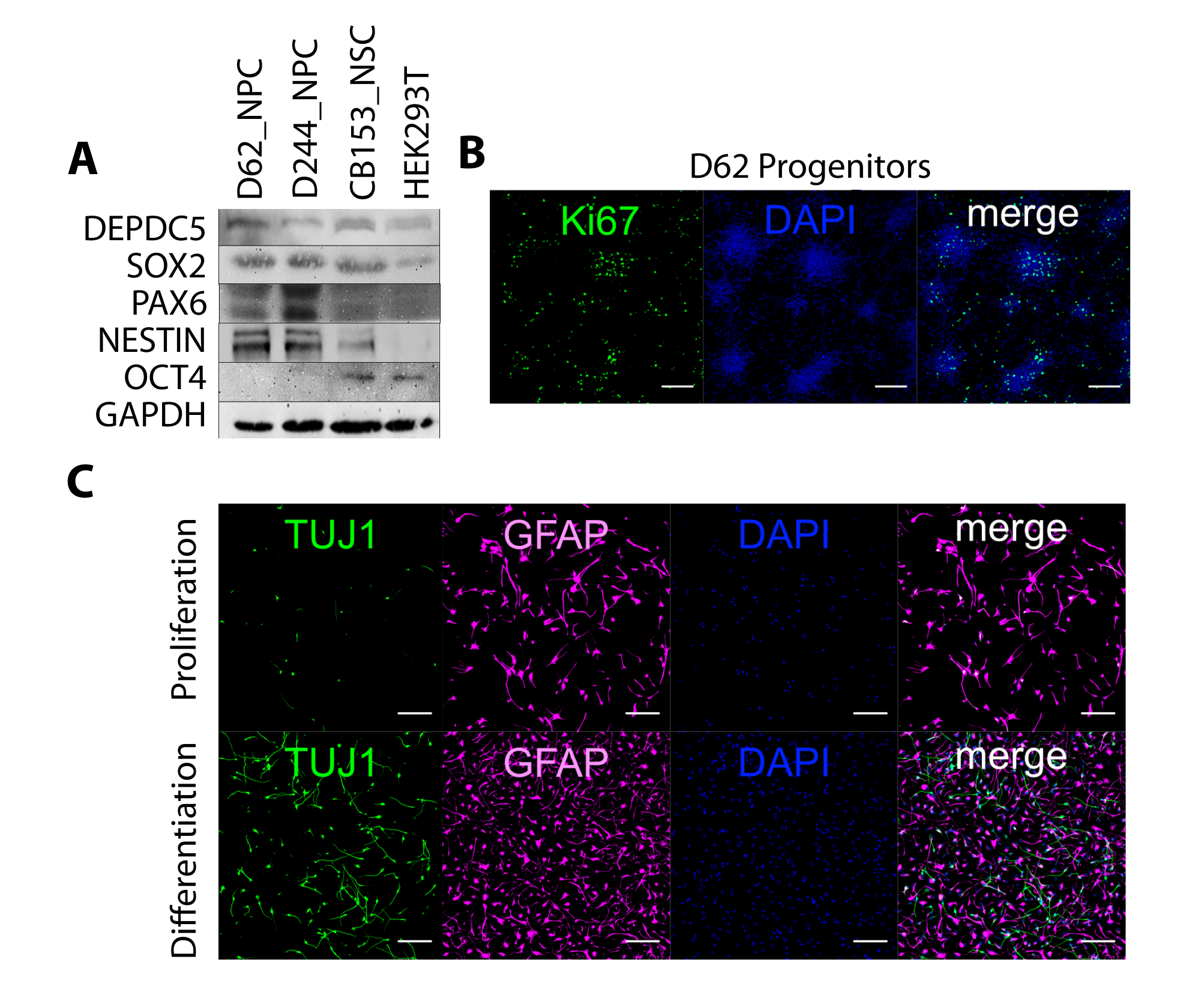


**Confirmation of phNPC phenotypes** (A) Western Blot of D62 human neural progenitor cells (hNPC used in this study), CB153 human neural stem cells and HEK293T human embryonic kidney cells stained for antibodies against DEPDC5, Sox2, Pax6, Nestin, Oct4 and GAPDH. (B) Immunocytochemistry of D62 progenitor cells stained against Ki67. Nuclei were stained with DAPI. (C) Comparison of immunocytochemical images of D62 progenitors during proliferation (top) and four weeks of differentiation (bottom) using markers for Tuj1 and GFAP. Nuclei were stained with DAPI. Scale bars: 100 μm. All stainings have been performed in biological replicates.

## Supplementary Figure S2:


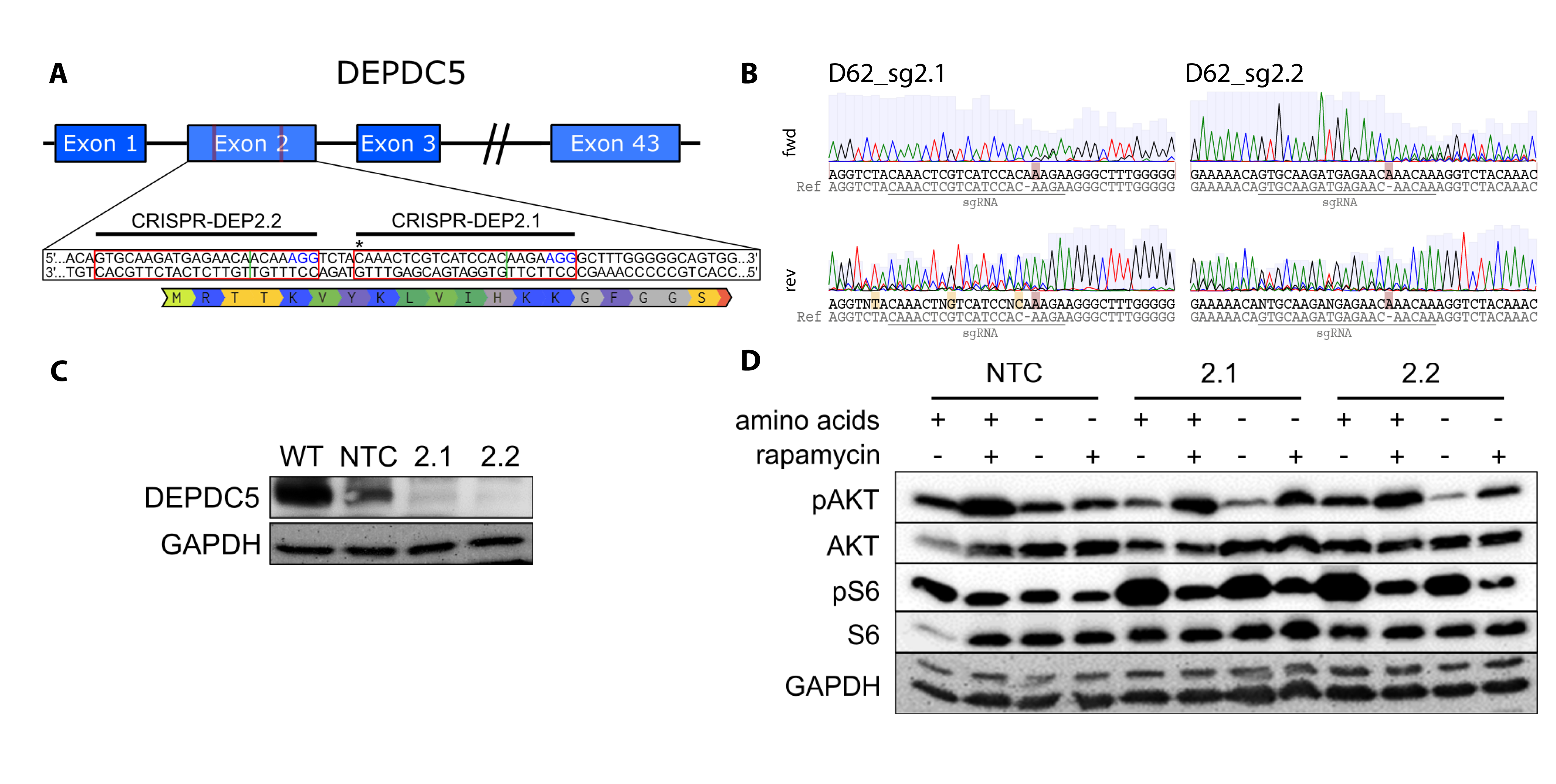


**CRISPR-Cas9-induced DEPDC5 mutations in phNPCs cause phosphorylation of S6 in amino acid-free media** (A) Design of sgRNAs targeting exon 2 of DEPDC5. Expected cut sites are labeled in green. PAM sequences are in blue. *Australian family mutation (B) Sanger sequencing of Dep2.1 and Dep2.2 knockout cell lines (bulk cultures). Trace qualities are labeled as bar graphs (purple). (C) Immunoblotting of DEPDC5 protein expression levels compared to GAPDH (control). (D) Western Blot of Dep2.1, Dep2.2 and NTC incubated in proliferation or amino acid free media with or without the addition of 100nM RAPA. Blots were cropped to show relevant bands. n=3. (AKT: protein kinase B, S6: ribosomal protein S6, p: phosphorylated)

## Supplementary Figure S3:

**
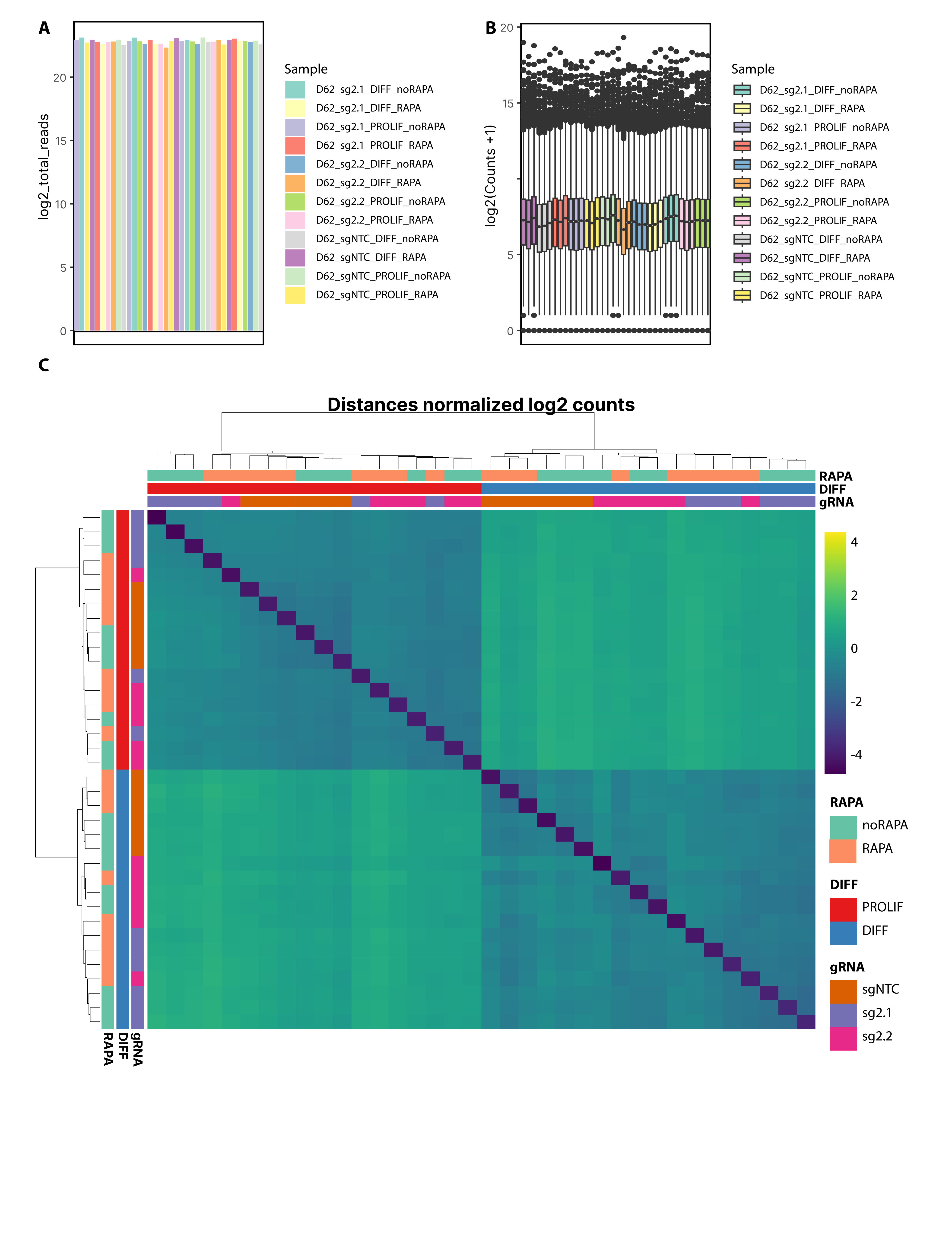
**

**Quality control of transcriptomic data of DEPDC5-KOs and NTC, during proliferation/differentiation, with and without RAPA**. (A) Total reads of RNA transcripts (average reads per sample 7,195,819 SD ± 345,374). (B) Average read per gene of NTC, 2.1, 2.2 under differentiation and proliferation. (C) Heatmap of unsupervised hierarchical clustering of distances normalized log2 counts between cell lines and conditions with main differences between proliferation and differentiation developmental stages.

## Supplementary Figure S4:


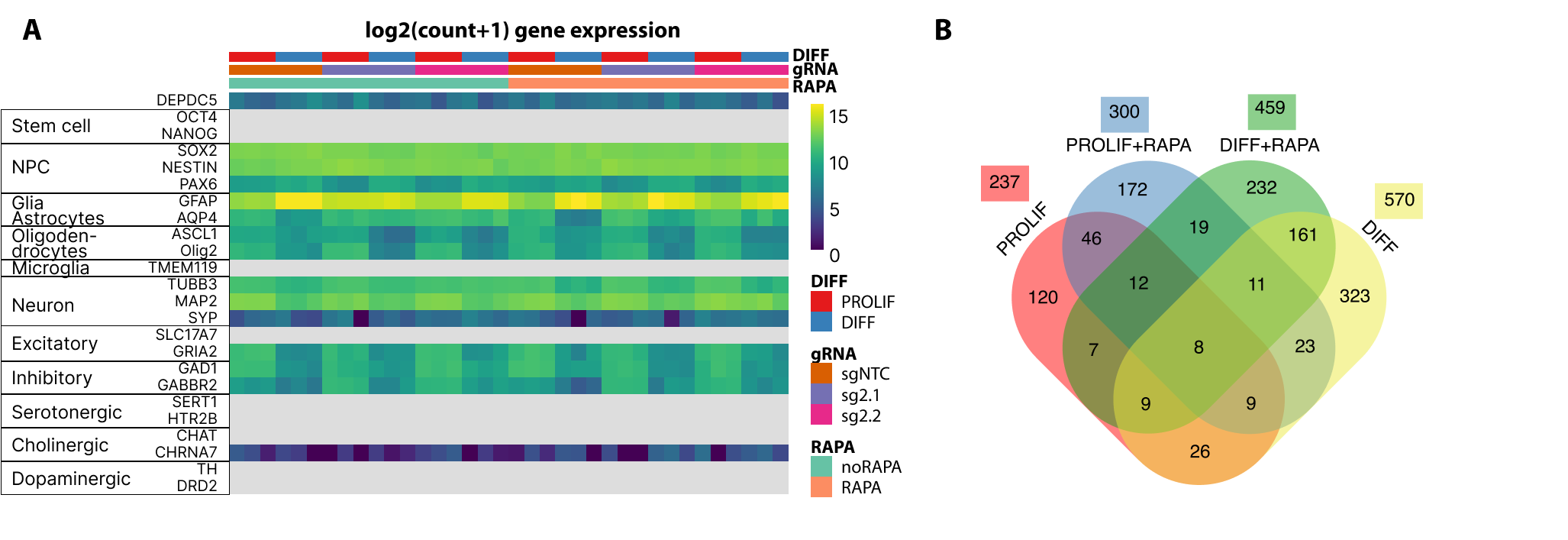


**Gene expression data of cell type specific markers and number of DEPDC5 KO-induced deregulated genes.** (A) RNA expression of different markers specific to neural stem cells, radial glia, astrocytes, immature neurons, oligodendrocytes, microglia, mature neurons and mature neuronal subtypes in DEPDC5 KO and NTC lines after 4 weeks of differentiation. (B) Venn diagram of number of deregulated genes associated with DEPDC5 KO. Colored ovals contain genes which were identified in proliferation (red), proliferation with RAPA (blue), differentiation (yellow) and differentiation with RAPA (green) conditions. Overlapping ovals show the total amount of genes which were recovered by RAPA administration while genes which were not attenuated are shown in the outer ovals.

## Supplementary Figure S5:


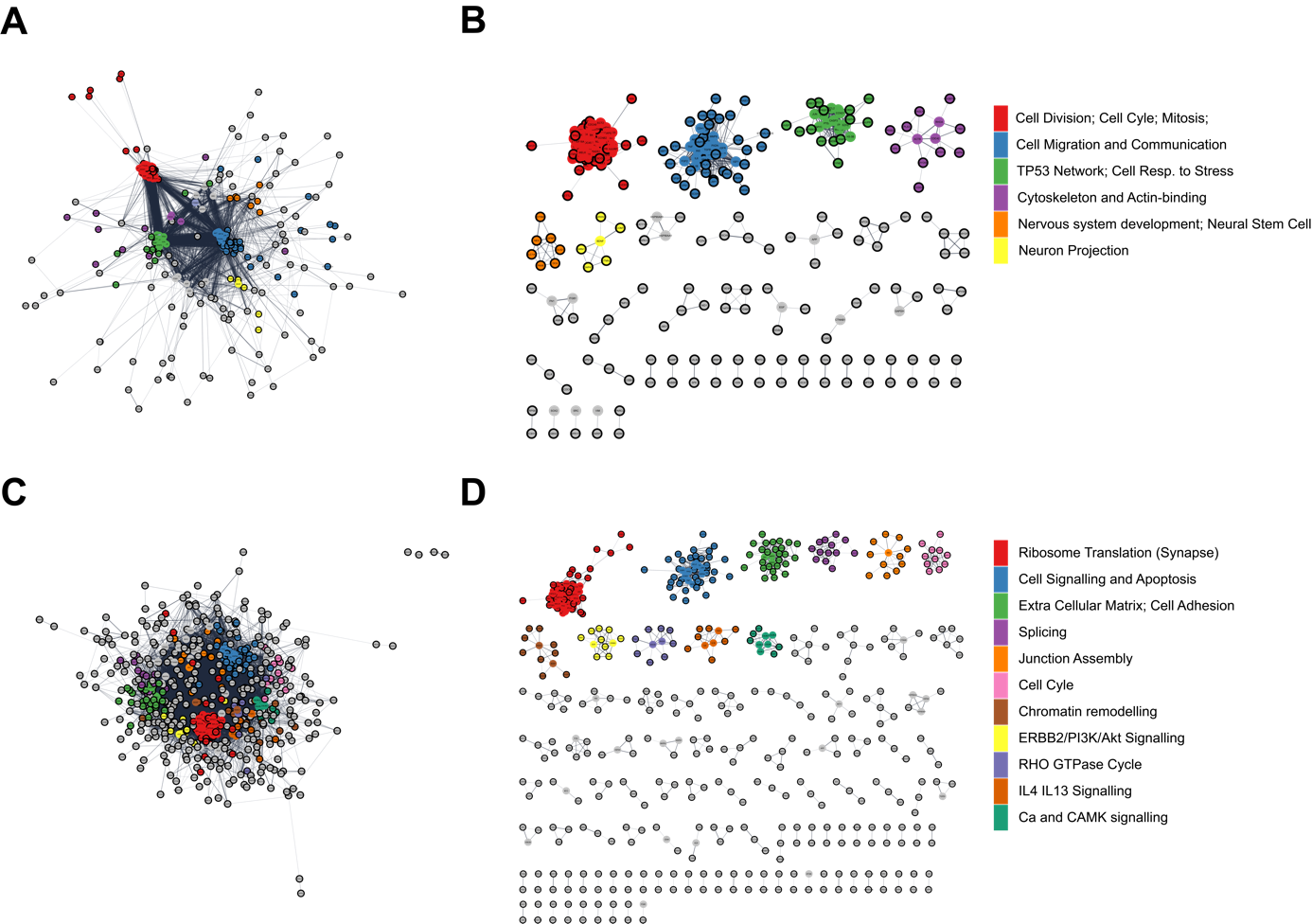


**Protein-Protein Interaction (PPI) Network analysis of DEX Genes**: PPI-Networks of DEX genes were extracted from the STRING Database using Cytoscape importing 100 additional interaction proteins. Subsequent MCL Cluster Analysis was performed with an inflation value of 4. Each identified DEX_PROLIF Genes (A-B) and DEX_DIFF genes (C-D) were analyzed independently. Differentially expressed genes identified in this study here are marked by a bold outline. Each identified cluster was tested for GO-term enrichment and labelled accordingly.

## Supplementary Figure S6:


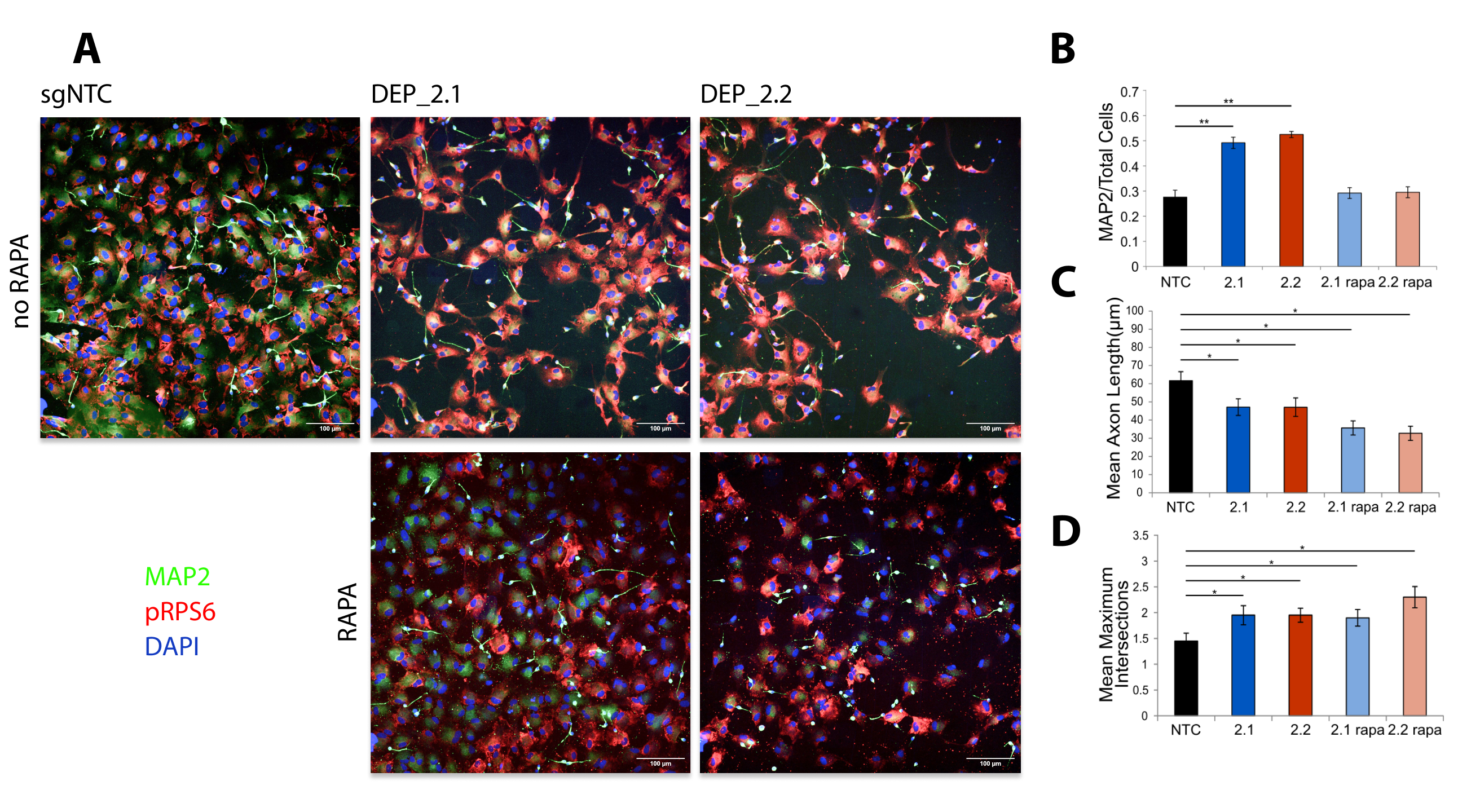


**Morphological analysis of DEPDC5 KO and RAPA rescue. NTC, Dep2.1 and 2.2 phNPCs without and with RAPA were differentiated for one month and subsequently stained for MAP2.** (A) Exemplary images of LSM used for morphological manual tracing. Dep2.1 and 2.2 phNPCs cultures show higher amounts of MAP2 positive neurons (GREEN) and pRPS6 expression (RED, validated in Western Blot Figure S2) Scale bar = 100µm. (B) Ratios of MAP2 positive neurons vs. total cell count. Sholl analysis for (C) mean axon length and (D) mean maximum intersections. Groups were compared with Students t-test compared to control. **p<0.01. Error bars represent ± SEM.

References

1. La Torre-Ubieta L de, Stein JL, Won H, Opland CK, Liang D, Lu D et al. The Dynamic Landscape of Open Chromatin during Human Cortical Neurogenesis. Cell 2018; 172(1-2):289-304.e18.

2. Stein JL, La Torre-Ubieta L de, Tian Y, Parikshak NN, Hernández IA, Marchetto MC et al. A quantitative framework to evaluate modeling of cortical development by neural stem cells. Neuron 2014; 83(1):69–86.

3. Rosen EY, Wexler EM, Versano R, Coppola G, Gao F, Winden KD et al. Functional genomic analyses identify pathways dysregulated by progranulin deficiency, implicating Wnt signaling. Neuron 2011; 71(6):1030–42.

4. Wexler EM, Rosen E, Lu D, Osborn GE, Martin E, Raybould H et al. Genome-wide analysis of a Wnt1-regulated transcriptional network implicates neurodegenerative pathways. Sci Signal 2011; 4(193):ra65.

5. Svendsen CN, ter Borg MG, Armstrong RJ, Rosser AE, Chandran S, Ostenfeld T et al. A new method for the rapid and long term growth of human neural precursor cells. Journal of Neuroscience Methods 1998; 85(2):141–52.

6. Ran FA, Hsu PD, Lin C-Y, Gootenberg JS, Konermann S, Trevino AE et al. Double nicking by RNA-guided CRISPR Cas9 for enhanced genome editing specificity. Cell 2013; 154(6):1380–9.

7. Dibbens LM, Vries B de, Donatello S, Heron SE, Hodgson BL, Chintawar S et al. Mutations in DEPDC5 cause familial focal epilepsy with variable foci. Nat Genet 2013; 45(5):546–51.

8. Stemmer M, Thumberger T, Del Sol Keyer M, Wittbrodt J, Mateo JL. CCTop: An Intuitive, Flexible and Reliable CRISPR/Cas9 Target Prediction Tool. PLoS One 2015; 10(4):e0124633.

9. Karra D, Dahm R. Transfection techniques for neuronal cells. J. Neurosci. 2010; 30(18):6171–7.

10. Dieterlen M-T, Wegner F, Schwarz SC, Milosevic J, Schneider B, Busch M et al. Non-viral gene transfer by nucleofection allows stable gene expression in human neural progenitor cells. Journal of Neuroscience Methods 2009; 178(1):15–23.

11. Kustikova OS, Wahlers A, Kuhlcke K, Stahle B, Zander AR, Baum C et al. Dose finding with retroviral vectors: correlation of retroviral vector copy numbers in single cells with gene transfer efficiency in a cell population. Blood 2003; 102(12):3934–7.

12. Bolger AM, Lohse M, Usadel B. Trimmomatic: a flexible trimmer for Illumina sequence data. Bioinformatics 2014; 30(15):2114–20.

13. Liao Y, Smyth GK, Shi W. The R package Rsubread is easier, faster, cheaper and better for alignment and quantification of RNA sequencing reads. Nucleic Acids Res 2019; 47(8):e47.

14. Langfelder P, Horvath S. WGCNA: an R package for weighted correlation network analysis. BMC Bioinformatics 2008; 9:559.

15. Ran X, Li J, Shao Q, Chen H, Lin Z, Sun ZS et al. EpilepsyGene: a genetic resource for genes and mutations related to epilepsy. Nucleic Acids Res 2015; 43(Database issue):D893-9.

16. Rubeis S de, He X, Goldberg AP, Poultney CS, Samocha K, Cicek AE et al. Synaptic, transcriptional and chromatin genes disrupted in autism. Nature 2014; 515(7526):209–15.

17. Iossifov I, O'Roak BJ, Sanders SJ, Ronemus M, Krumm N, Levy D et al. The contribution of de novo coding mutations to autism spectrum disorder. Nature 2014; 515(7526):216–21.

18. Voineagu I, Wang X, Johnston P, Lowe JK, Tian Y, Horvath S et al. Transcriptomic analysis of autistic brain reveals convergent molecular pathology. Nature 2011; 474(7351):380–4.

19. Darnell JC, van Driesche SJ, Zhang C, Hung KYS, Mele A, Fraser CE et al. FMRP stalls ribosomal translocation on mRNAs linked to synaptic function and autism. Cell 2011; 146(2):247–61.

20. Grabole N, Zhang JD, Aigner S, Ruderisch N, Costa V, Weber FC et al. Genomic analysis of the molecular neuropathology of tuberous sclerosis using a human stem cell model. Genome Med 2016; 8(1):94.

21. Martin KR, Zhou W, Bowman MJ, Shih J, Au KS, Dittenhafer-Reed KE et al. The genomic landscape of tuberous sclerosis complex. Nat Commun 2017; 8:15816.

22. Pinto D, Delaby E, Merico D, Barbosa M, Merikangas A, Klei L et al. Convergence of genes and cellular pathways dysregulated in autism spectrum disorders. Am J Hum Genet 2014; 94(5):677–94.

23. Parikshak NN, Luo R, Zhang A, Won H, Lowe JK, Chandran V et al. Integrative functional genomic analyses implicate specific molecular pathways and circuits in autism. Cell 2013; 155(5):1008–21.

24. Fromer M, Roussos P, Sieberts SK, Johnson JS, Kavanagh DH, Perumal TM et al. Gene expression elucidates functional impact of polygenic risk for schizophrenia. Nat Neurosci 2016; 19(11):1442–53.

25. Cocchi E, Drago A, Serretti A. Hippocampal Pruning as a New Theory of Schizophrenia Etiopathogenesis. Mol Neurobiol 2016; 53(3):2065–81.

26. Raudvere U, Kolberg L, Kuzmin I, Arak T, Adler P, Peterson H et al. g:Profiler: a web server for functional enrichment analysis and conversions of gene lists (2019 update). Nucleic Acids Res 2019; 47(W1):W191-W198.

27. Yousaf A, Duketis E, Jarczok T, Sachse M, Biscaldi M, Degenhardt F et al. Mapping the genetics of neuropsychological traits to the molecular network of the human brain using a data integrative approach; 2018. ( vol 5).

28. Kang HJ, Kawasawa YI, Cheng F, Zhu Y, Xu X, Li M et al. Spatio-temporal transcriptome of the human brain. Nature 2011; 478(7370):483–9.
